# Shared and disease-specific pathways in frontotemporal dementia and Alzheimer’s and Parkinson’s diseases

**DOI:** 10.1038/s41591-025-03833-1

**Published:** 2025-07-15

**Authors:** Muhammad Ali, Buddhiprabha Erabadda, Yike Chen, Ying Xu, Katherine Gong, Menghan Liu, Alexa Pichet Binette, Jigyasha Timsina, Daniel Western, Chengran Yang, Gyujin Heo, Jacob W. Vogel, Betty M. Tijms, Varsha Krish, Farhad Imam, The Global Neurodegeneration Proteomics Consortium (GNPC), Muhammad Ali, Yike Chen, Ying Xu, Menghan Liu, Alexa Pichet Binette, Jigyasha Timsina, Gyujin Heo, Jacob W. Vogel, Betty M. Tijms, Varsha Krish, Farhad Imam, Oskar Hansson, Laura Winchester, Carlos Cruchaga, Oskar Hansson, Laura Winchester, Carlos Cruchaga

**Affiliations:** 1 Department of Psychiatry, Washington University School of Medicine, St. Louis, MO USA; 2 NeuroGenomics and Informatics Center, Washington University School of Medicine, St. Louis, MO USA; 3 Department of Psychiatry, University of Oxford, Oxford, UK; 4 Clinical Memory Research Unit, Department of Clinical Sciences Malmö, Lund University, Lund, Sweden; 5 Department of Clinical Sciences Malmö, SciLifeLab, Lund University, Lund, Sweden; 6 Alzheimer Center Amsterdam, Neurology, Vrije Universiteit Amsterdam, Amsterdam UMC location VUmc, Amsterdam, The Netherlands; 7 Amsterdam Neuroscience, Neurodegeneration, Amsterdam, The Netherlands; 8 Gates Ventures, Seattle, WA USA; 9 Eli Lilly, Stockholm, Sweden; 10 Department of Neurology, Washington University School of Medicine, St. Louis, MO USA; 11 Hope Center for Neurological Disorders, Washington University, St. Louis, MO USA; 12 Department of Genetics, Washington University School of Medicine, St. Louis, MO USA

**Keywords:** Molecular neuroscience, Alzheimer's disease, Diagnostic markers

## Abstract

Neurodegenerative diseases (NDs), such as Alzheimer’s disease (AD), Parkinson’s disease (PD) and frontotemporal dementia (FTD), exhibit distinct yet overlapping pathological mechanisms. Leveraging large-scale plasma proteomics data from the Global Neurodegeneration Proteomics Consortium, we analyzed 10,527 plasma samples (1,936 AD, 525 PD, 163 FTD, 1,638 dementia and 6,265 controls) to identify disease-specific and shared proteins across NDs. We identified 5,187 proteins significantly associated with AD, 3,748 with PD and 2,380 with FTD that revealed both common and divergent proteomic signatures, which were confirmed by multiple analytical approaches and orthogonal validation. PD and FTD showed the highest overlap (*r*^2^ = 0.44) and AD and PD the least (*r*^2^ = 0.04). Immune system, glycolysis, and matrisome-related pathways were enriched across all NDs, while disease-specific pathways included apoptotic processes in AD, endoplasmic reticulum–phagosome impairment in PD and platelet dysregulation in FTD. Network analysis identified key upstream regulators (RPS27A in AD, IRAK4 in PD and MAPK1 in FTD) potentially driving these proteomic changes. These findings reveal distinct and shared mechanisms across NDs, highlighting potential regulatory proteins and pathways for diagnostic and therapeutic strategies in neurodegeneration.

## Abstract (web summary)

Large-scale plasma proteomics of 10,527 samples from the Global Neurodegeneration Proteomics Consortium identified shared and disease-specific pathways in Alzheimer’s disease, Parkinson’s disease and frontotemporal dementia.

## Main

Alzheimer’s disease (AD), Parkinson’s disease (PD) and frontotemporal dementia (FTD) are among the most prevalent neurodegenerative diseases (NDs), each characterized by distinct yet overlapping molecular and pathological features. Although AD, PD and FTD are often defined by specific pathological hallmarks such as amyloid-beta (Aβ) and tau in AD^1^, alpha-synuclein in PD^2^ and tau, TDP-43 or FUS in FTD^3^, there is clinical and neuropathological overlap across these diseases. In total, 19–57% of AD cases exhibit TDP-43 pathology^4^, and α‐synuclein positivity is observed in approximately 20–30% of patients with AD^5^. This clinical and pathological intersection complicates differential diagnosis and hinders the development of effective treatments, underscoring the need for a comparative molecular analysis to elucidate shared and disease-specific mechanisms of neurodegeneration.

Plasma proteomics, in particular, provide a minimally invasive approach to studying systemic disease signatures and capturing peripheral changes associated with neurodegeneration. High-throughput platforms such as mass spectrometry and affinity-based methods (for example, SomaScan or Olink) have enabled the quantification of hundreds to thousands of proteins, revealing disease-associated alterations. For example, studies of AD^6–9^, including our own^10^, have identified proteins involved in immune, lipid, cell proliferation and chemotaxis pathways. Similarly, plasma proteomics studies in PD have identified proteins involved in complement activation, neuroinflammation and platelet degranulation^11,12^. In contrast, plasma-based studies in FTD are limited, with small cohorts and fewer proteins measured, identifying only up to 13 significantly altered proteins^13,14^. This scarcity of data limits the exploration of FTD-specific biological pathways, emphasizing the need for larger, well-characterized cohorts to identify proteomic alterations and pathways implicated in FTD.

Network-based analyses of proteomic data have considerably advanced our understanding of NDs by identifying key proteins and pathways involved in disease. For instance, multiple studies on AD brain tissues uncovered modules of co-expressed proteins associated with RNA splicing, synaptic function and inflammation^15^. Similarly, proteomic studies have identified dysregulated protein networks related to mitochondrial function, Wnt signaling and oxidative stress in PD^12^ and synaptic function, immune activation and RNA processing in FTD^16^.

Despite these advances, there remains a need for plasma proteomic studies that go beyond a single-disease focus to include multiple NDs with large sample sizes, enabling the identification of shared and disease-specific molecular signatures. Moreover, although protein-level association analyses are commonly conducted, systematic evaluations of proteomic correlations across diseases and in-depth analyses of converging and diverging molecular mechanisms are still lacking. Furthermore, the regulatory pathways between proteins, which may reveal upstream modulators driving disease pathogenesis, remain underexplored.

In this study, we leveraged large-scale plasma proteomics data from the Global Neurodegeneration Proteomics Consortium (GNPC) to investigate proteomic associations with AD, PD and FTD. We performed differential abundance analysis to identify disease-associated proteins, followed by effect size correlation analyses, to assess the degree of molecular convergence and divergence across these disorders. Pathway and network analyses were conducted to determine biological processes that are commonly or selectively dysregulated in each disease as well as key upstream regulators that may drive neurodegenerative processes.

## Results

### Patients, proteomic data and study design

This study used a large, cross-sectional plasma proteomics dataset from GNPC version 1 containing samples from 23 independent contributing sites. The broader GNPC resource includes a total of 31,111 samples from 21,979 individuals diagnosed with a range of diseases, including neurodegenerative disorders, depression, diabetes, stroke and several others. Among these, 1,638 individuals were assigned a dementia diagnosis based on a global Clinical Dementia Rating (CDR)^17^ greater than 0.5 or a Mini-Mental State Examination (MMSE)^18^ score lower than 19, in the absence of a confirmed clinical diagnosis. For this study, we included individuals with clinical diagnoses of AD, FTD and PD and cognitively normal controls. We defined controls as those who are cognitively normal, with a CDR of 0 and an MMSE score of 24 or higher, and AD as those with clinical diagnosis of AD and CDR above 0. Similarly, patients with PD and patients with FTD were clinically diagnosed. The final dataset included 10,527 cross-sectional samples from 16 contributor sites, including 1,936 AD, 525 PD, 163 FTD, 1,638 dementia and 6,265 controls (Table 1).

**Table 1:** Demographics information of participants at the time of plasma draw

Clinical disease	Sample size (*N*)	Female (%)	Age mean (s.d.)	APOE4^+^ (%)
**AD**	1,936	55	77 (9)	44
**PD**	525	50	71 (9)	17
**FTD**	163	16	63 (10)	10
**Dementia**	1,638	58	72 (13)	33
**CO**	6,265	53	70 (13)	23

Basic demographic information of plasma proteomics data in the GNPC. For each ND (AD, PD and FTD), we report the total sample size (*N*), percentage of females and mean age and its s.d. CO, controls.

**Fig. 1:**
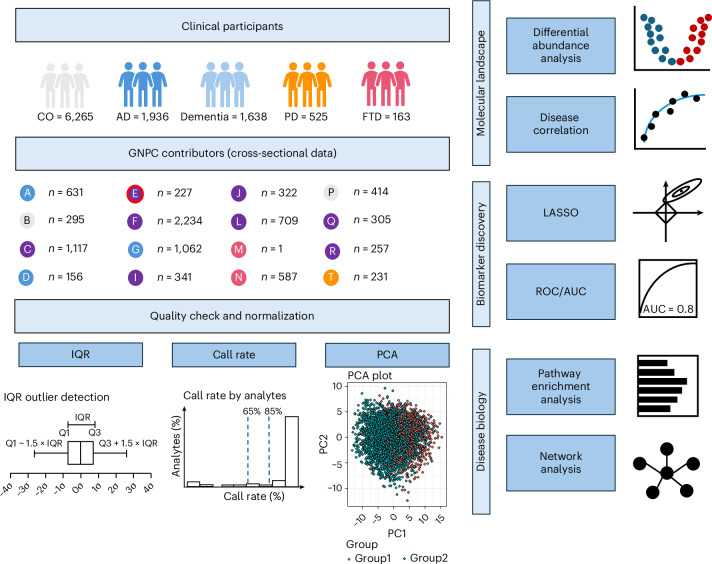
A framework for mapping human plasma proteome in neurodegeneration. Plasma samples were collected from 1,936 AD, 525 PD, 163 FTD and 6,265 cognitively normal control participants and profiled using the SomaScan 7K platform. These proteomic data are available in the GNPC from 16 independent contributor sites. The Site E (marked by red boundary) collected blood in sodium citrate tubes, whereas all remaining sites collected plasma samples in EDTA tubes. Stringent quality control and *z*-score normalization were performed to harmonize these datasets and remove the batch effects (*σ* in the IQR boxplot denotes standard deviation from the mean). Differential protein abundance analysis was performed to identify proteins associated with each disease, and pairwise comparisons of protein effect size across disease pairs were assessed to map the proteomic landscape of neurodegeneration. Machine learning approaches were leveraged to identify disease-specific prediction models. Pathway, cell type enrichment and network analyses were conducted to understand underlying disease biology. CO, controls; PC, principal component; PCA, principal component analysis.

Proteomic profiling was conducted using the SomaScan assay version 4.1, which quantified 7,595 aptamers targeting 6,386 unique human proteins. In total, 7,289 aptamers passed quality control (Methods). To identify disease-associated proteins, we performed linear regression analyses comparing each disease group (AD, PD and FTD) to the control group. The models were adjusted for age, sex and the first two proteomic principal components to account for potential confounding factors. Proteins passing a false discovery rate (FDR) threshold of less than 0.05 were considered significant. Next, we evaluated the pairwise correlation of effect sizes for significant proteins to assess molecular similarities and differences across NDs. Additionally, we conducted cell type enrichment, pathway and network analyses to identify biological processes and key proteins that are commonly or uniquely dysregulated in each disease (Fig. 1). This comprehensive approach provides a systematic understanding of the proteomic landscape underlying AD, PD and FTD.

### Differences in plasma proteomes across neurodegeneration

**Fig. 2:**
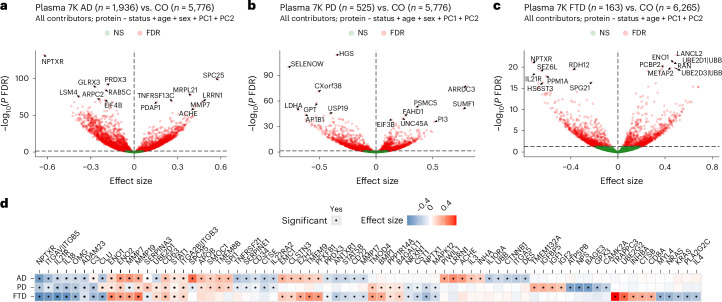
Plasma proteomic alterations in AD, PD and FTD compared to controls. Volcano plots displaying proteins with significantly increased and decreased abundance in AD (**a**), PD (**b**) and FTD (**c**) compared to controls (CO). Each point represents a protein, with the *x* axis showing the effect size and the *y* axis showing the FDR from the linear regression model. The red points indicate proteins with significant differential abundance (FDR < 0.05); the green points represent non-significant proteins. Key proteins with notable changes are labeled with the protein name. The dashed lines on the *x* axis denote the significance threshold, where proteins to the right indicate increased abundance and those to the left indicate decreased abundance in diseased samples in comparison to CO. **d**, Heatmap displaying the effect size of key significantly altered proteins across AD, PD and FTD. The proteins are ordered based on their significant associations with all three diseases, AD and PD, AD and FTD, PD and FTD and the proteins uniquely associated with AD, PD and FTD, respectively. Blue and red colors in the heatmap indicate decreased and increased abundance, respectively. Black dots inside the squares indicate statistically significant associations (FDR < 0.05). NS, not significant.

Among the more than 7,000 protein aptamers analyzed, 5,187 (71%) were significantly associated with AD, 3,748 (51%) with PD and 2,380 (33%) with FTD (Fig. 2 and Supplementary Table 1).

**Extended Data Fig. 1:**
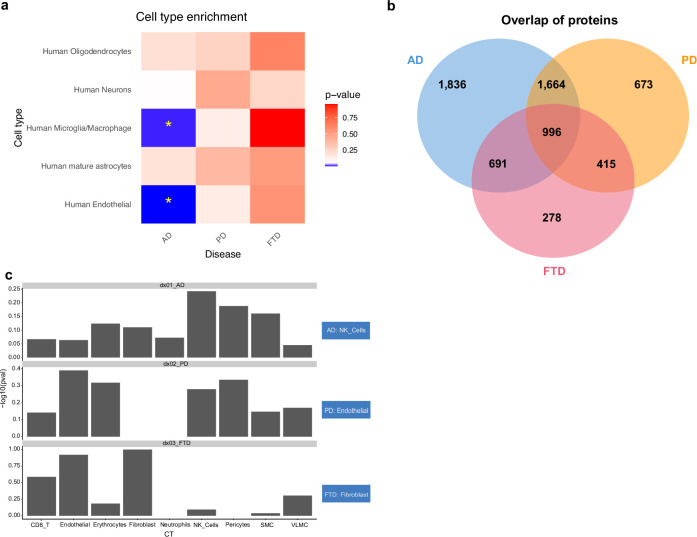
Overlap and cell type enrichment of disease-associated proteins. **a**, Heatmap representing the enrichment of disease-associated proteins from each disease (AD, PD, and FTD) in five different human brain cell types (oligodendrocytes, neurons, microglia/macrophages, mature astrocytes, and endothelial cells). The significance of enrichment (hypergeometric *p-value*) was computed using hypergeometric test. The significant enrichment (*p* < 0.05) of disease-associated proteins with a particular cell type is denoted by * in the heatmap square. **b**, Overlap of significant (FDR < 0.05) disease-associated proteins across AD, PD, and FTD. **c**, Enrichment of AD-, PD-, and FTD-associated proteins in human blood cell types. No cell type reached statistical significance for enrichment, the top-ranked cell type varied by disease, with natural killer (NK) cells ranking highest in AD, endothelial cells in PD, and fibroblasts in FTD.

**Extended Data Fig. 2:**
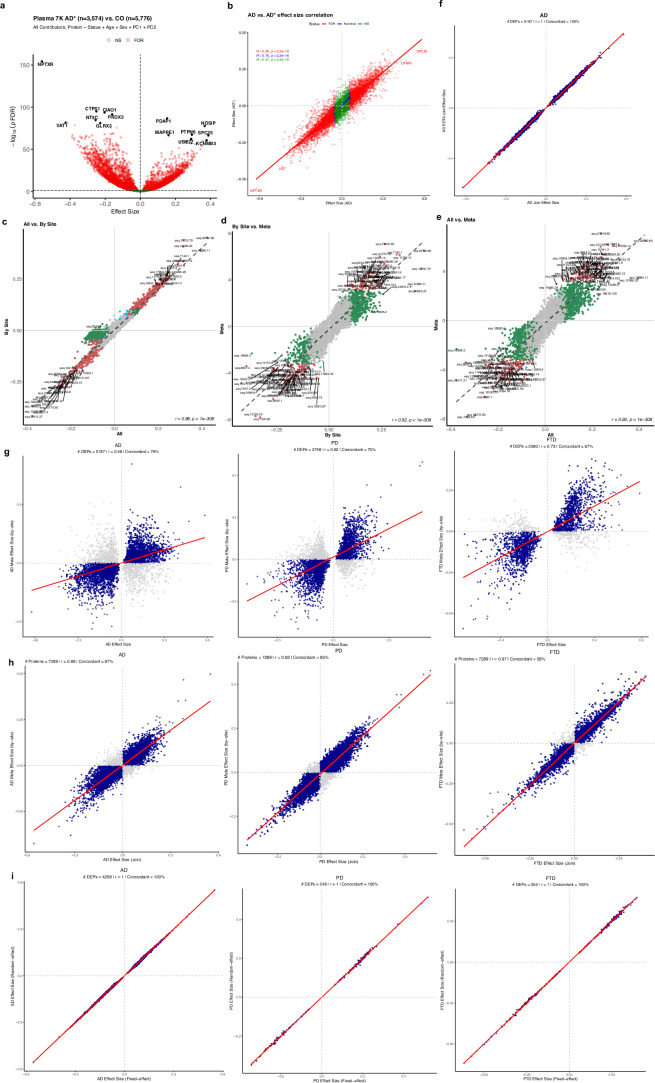
Sensitivity analyses. To rule out the possibility that the large number of differentially abundant proteins in AD is an artifact of the analyses, due to data harmonization across sites, bias due to site or any other hidden problem, we conducted several additional analyses (e.g. analyses using raw proteomic values, inclusion of site in regression model, and joint vs. meta-analysis) to demonstrate the robustness of our analyses. We demonstrate that different QC approaches, either doing QC by site or all samples together do not lead to considerably different results. Additionally, the inclusion or exclusion of sodium citrate samples does not change the findings. Furthermore, the joint analyses do not lead to any batch or artifactual results as the effect sizes of the joint analyses are highly correlated with those of the meta-analyses. Moreover, the joint analyses that show similar effect sizes to that of the meta-analyses provide more statistical power by reducing the confidence interval of the estimates. **a-b**, Sensitivity analysis performed using extended AD and dementia patients. **a**, Volcano plot visualizing proteins significantly increased (right side of dashed vertical line on x-axis) or decreased (left side of dashed vertical line on x-axis) in AD and dementia patients in comparison to cognitively normal controls. The dotted line on the y-axis represents the significance threshold (FDR < 0.05). In the GNPC version 1.3, 1,638 individuals have been diagnosed with dementia, based on a Clinical Dementia Rating (CDR) greater than 0.5 or a Mini-Mental State Examination (MMSE) score below 19, but do not have a confirmed final diagnosis. **b**, A scatterplot of correlation in effect size from the AD vs. CO analysis (included in the main results) and AD and Dementia vs. CO analysis (sensitivity analysis). Red and blue dots and regression lines represent proteins that passed FDR (FDR < 0.05) and nominal (*p* < 0.05) significance in the main AD vs. CO analysis, and green dots represent proteins that are non-significant (*p* > 0.05). We observed a strong correlation (Pearson *r*^2^ = 0.93) in effect size between the main and sensitivity analyses. **c-e**, Pairwise comparisons of effect size estimates from three models. **c**, Joint analysis using study-wide Z-scores (All). **d**, Joint analysis using z-score calculated by site (By Site). **e**, Meta-analyses using z-score by site (Meta). Each point represents protein, with effect estimates shown for the corresponding models. Points are color-coded based on significance in the respective models: grey indicates not significant in either model, green indicates significance in the x-axis model only, blue in the y-axis model only, and red indicates significance in both models. Dashed lines indicate linear regression fits with shaded 95% confidence intervals. Spearman correlation coefficients (r) and associated P values are displayed in each panel. **f**, Effect size correlation of the analyses with and without the site with Citrate. Effect size estimates were highly consistent, with 100% directional concordance and a Pearson correlation coefficient of *r*^2^ = 1. Scatter plot of effect size EDTA joint analysis (x-axis) for differentially expressed proteins versus EDTA + Citrate joint analysis effect sizes (y-axis) across AD. The number of differentially expressed proteins is 5,187. Each dot represents a protein, with blue indicating concordant direction and grey indicating discordant direction between the two analyses. The red line indicates linear regression fit. **g-h**, Correlation of effect sizes between by-site meta-analysis and joint analysis. To perform a head-to-head comparison of the meta-analyses vs the joint analyses, we initially compare the results of a joint analyses with those of the meta-analyses where only the sites with cases and controls for a specific disease are included. In this way the same samples are included in both the meta-analyses and the joint analyses. When doing these analyses, we found high correlation (*r*^2^ > 0.88) in the effect size, and even higher concordance rate in direction of effect sizes across both analyses (>95%). This correlation further increases if we focus only on the proteins that passed FDR correction in the joint analysis. Scatter plots of effect size joint analysis (x-axis) for differentially expressed proteins vs. by-site meta-analysis effect sizes (y-axis) across three diseases: AD, PD, (FTD. Each dot represents a protein, with blue indicating concordant direction and grey indicating discordant direction between the two analyses. The red line indicates linear regression fit. **g**, Represents all proteins. **h**, Only proteins that pass FDR significance. **i**, Correlation of effect sizes between joint analysis (Fixed effect) and joint analysis (Random effect). Scatter plots of effect size joint analysis fixed effect (x-axis) for differentially expressed proteins vs. joint analysis random effect (y-axis) across three diseases: AD, PD, and FTD. Each dot represents a protein, with blue indicating concordant direction and grey indicating discordant direction between the two analyses. The red line indicates linear regression fit. The results from the two approaches were highly consistent. Specifically, the correlation of effect sizes is quite high (*r*^2^ > 0.98), and there is minimal changes in the proteins that pass FDR, suggesting that the random vs fixed effect has low impact on the overall associations.

The large sample size and high statistical power of this study enabled the validation of many known disease-associated proteins and biomarkers while also uncovering novel proteins not previously implicated in neurodegeneration through plasma proteomics. In AD, several established biomarkers exhibited significant associations, including 14-3-3 protein YWHAH (*β* = 0.10, *P* = 5.9 × 10^−26^), SMOC1 (*β* = 0.20, *P* = 1.6 × 10^−21^) and PPP3R1 (*β* = 0.10, *P* = 4.3 × 10^−6^)^10,19^. We also validated additional proteins reported in a recent large-scale AD proteomic plasma study: NPTXR (*β* = −0.62, *P* = 4.9 × 10^−136^), SPC25 (*β* = 0.58, *P* = 7.7 × 10^−99^), LRRN1 (*β* = 0.47, *P* = 1.1 × 10^−71^), MAPT (*β* = 0.07, *P* = 4.7 × 10^−3^) and ACHE (*β* = 0.41, *P* = 4.3 × 10^−62^)^10^. Compared to previous plasma proteomic studies, we also found novel proteins, such as PRDX3 (*β* = −0.19, *P* = 3.8 × 10^−95^), ENO2 (*β* = 0.41, *P* = 7.5 × 10^−51^), UBB (*β* = −0.23, *P* = 1.3 × 10^−43^), CTNNB1 (*β* = −0.30, *P* = 2.3 × 10^−43^), PSMB10 (*β* = −0.27, *P* = 6.5 × 10^−42^), DSG1 (*β* = −0.30, *P* = 1.6 × 10^−41^), MMP19 (*β* = 0.35, *P* = 6.1 × 10^−40^), RPS27A (*β* = −0.06, *P* = 2.2 × 10^−5^) and TAX1BP1 (*β* = 0.08, *P* = 3.2 × 10^−3^). We also identified several apoptotic proteins uniquely associated with AD, including desmogleins (DSG1, DSG2 and DSG3) and caspases (CASP3, CASP7 and CASP8). Notably, AD-associated proteins revealed significant enrichment in human endothelial (*P* = 9.3 × 10^−3^) and microglial/macrophage (*P* = 0.01) cell types (Extended Data Fig. 1). Blood cell-type enrichment identified natural killer cells as highly enriched in AD, endothelial cells in PD and fibroblasts in FTD (Extended Data Fig. 1c). A similar number of differentially abundant proteins were identified when including any individual with AD or unspecified dementia, with a strong effect size correlation (Pearson’s *r*^2^ = 0.93, *P* < 1.0 × 10^−300^; Extended Data Fig. 2a,b and Supplementary Table 2).

In PD, numerous proteins associated with protein degradation and ubiquitination were identified: HGS (*β* = −0.35, *P* = 2.1 × 10^−118^), ARRDC3 (*β* = 0.80, *P* = 8.9 × 10^−82^), PSMC5 (*β* = 0.36, *P* = 1.4 × 10^−56^) and USP19 (*β* = −0.41, *P* = 1.1 × 10^−48^) as well as various proteasomes (PSME1, PSMD11 and PSMB4) and kinases (MAPK11, MAPK13 and IRAK4), reinforcing the critical role of the ubiquitin–proteasome system and improper protein phosphorylation in PD pathogenesis^20^. Well-known PD-related proteins, including PARK7 (*β* = −0.14, *P* = 2.7 × 10^−23^), PRKN (*β* = −0.09, *P* = 6.4 × 10^−3^), LRRK2 (*β* = −0.13, *P* = 2.7 × 10^−4^), FOXO3 (*β* = −0.05, *P* = 2.2 × 10^−2^) and SNCA (*β* = 0.05, *P* = 2.5 × 10^−2^), were also associated with PD. Similarly, in FTD, key proteins implicated in tauopathies and neurofilament dynamics were identified: MAPT (*β* = −0.32, *P* = 3.1 × 10^−5^) and NEFL (*β* = 0.28, *P* = 2.3 × 10^−4^). Notably, proteins linked to the ubiquitin–proteasome system, such as UBE2D1 | UBB (*β* = 0.61, *P* = 9.7 × 10^−26^), UBE2D3 | UBB (*β* = 0.49; *P* = 1.8 × 10^−23^) and PSMB3 (*β* = 0.26, *P* = 3.3 × 10^−4^), were also identified, further supporting the established role of impaired protein degradation in FTD pathogenesis^21^. Additionally, proteins such as ENO1 (*β* = 0.47, *P* = 2.6 × 10^−25^) and NPTXR (*β* = −0.72, *P* = 5.8 × 10^−25^), involved in glycolysis and synaptic functions, respectively, were also associated with FTD, highlighting metabolic dysregulation and synaptic dysfunction^22^. Finally, several interleukin (for example, IL-20RA, IL-2 and IL-1F10) and cathepsin (CTSC, CTSH and CTSV) proteins were associated with FTD.

**Extended Data Fig. 3:**
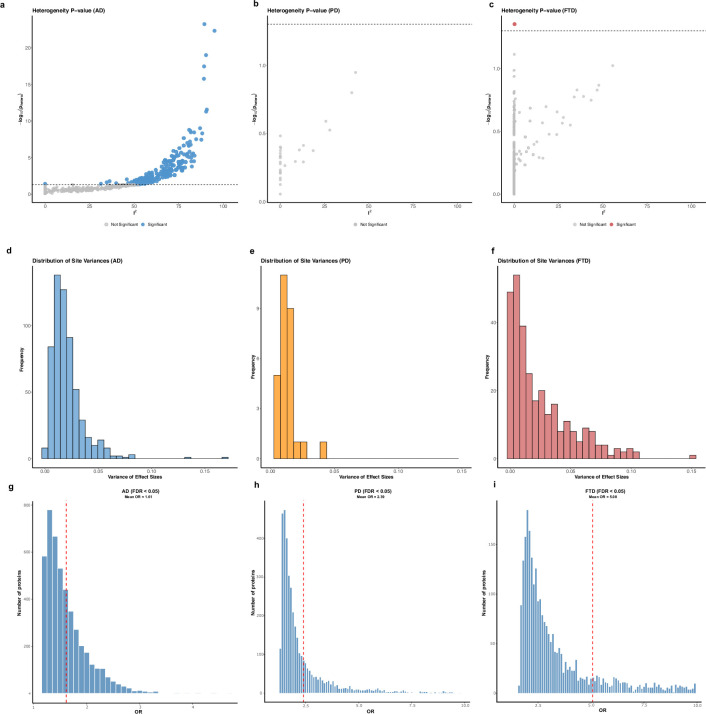
Assessment of heterogeneity (I^2^) and histograms of odds ratios for FDR < 0.05 proteins. **a-c**, The scatter plot examines the relationship between heterogeneity, as measured by I^2^, and the transformed heterogeneity p-value -log_10_(p_hetero_). Points above the dashed line are colored and represent significant heterogeneity (p < 0.05), while grey points below the line are not significant. The x-axis shows the percentage of variance caused by real differences, not random error. **d-f**, These histograms display the distribution of site-level variances for proteins in each disease dataset. The x-axis shows the variance of effect sizes across sites, and the y-axis indicates how many proteins fall within each variance range. **g–i**, panels show the distribution of ORs for **g**, AD, **h**, PD, and **i**, FTD. Red dashed lines indicate the mean symmetric OR in each disease group.

**Extended Data Fig. 4:**
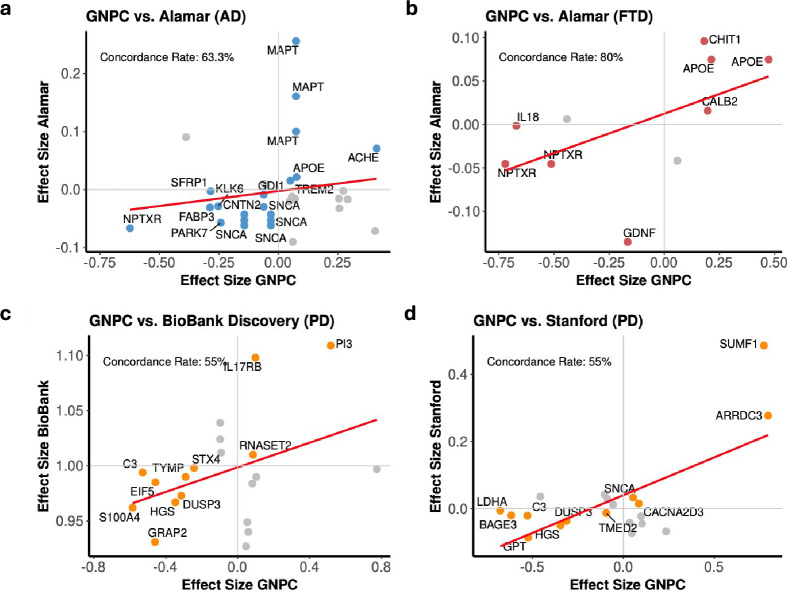
Comparison of significant protein concordance across orthogonal platforms and external datasets. **a**, Of the shared aptamers, we selected the top 15 and bottom 15 significant proteins of selected by GNPC, out of the 30 proteins, 19(63.3%) of them are of the same direction in Alamar, colored by the blue point. **b**, Same strategy was also applied to the FTD comparison. Due to the sample size limitation, we selected top 5 and bottom 5 significant proteins in GNPC, 8 out of 10 (80%) are of same direction of effect size in Alamar, colored by pink. **c**, Comparison with the work by Yi-Han et al.: Their work was conducted on the Olink platform, we analyzed the top 10 and bottom 10 significant shared proteins in GNPC based on FDR values. It demonstrated concordance rates of 55% in Discovery (UK Biobank). **d**, Comparison with the work by Rutledge et al. (SomaScan Stanford-5x): Among the shared top 10 and bottom 10 proteins in GNPC, a concordance rate of 55% was observed. In summary, these analyses underscore the overlap and consistency of protein directional effects across platforms and diseases, offering valuable insights into the reproducibility and cross-platform validation of our findings.

To evaluate consistency of protein associations across different cohorts, we assessed site-level heterogeneity, finding that most FDR-significant proteins showed low variance (<0.05; Extended Data Fig. 3a–f). To put into context the strength of the association for the identified proteins, we computed odds ratios comparing extreme tertiles, yielding average odds ratios of 1.61 for AD, 2.39 for PD and 5.08 for FTD (Extended Data Fig. 3g–i and Supplementary Table 3). Although no external study currently matches the sample size of GNPC for a comprehensive orthogonal validation, we assessed the consistency of our findings using independent proteomic datasets from the UK Biobank (Olink platform)^23^, the Knight ADRC (Alamar platform)^24^ and the Stanford ADRC (SomaScan platform)^11^ cohorts (Extended Data Fig. 4). In AD, 63.3% of the selected proteins showed consistent directional effects in the Alamar data. For FTD, 80% of proteins demonstrated concordant directions. In PD, directional concordance was observed in 55.0% of proteins across both the Olink and SomaScan platforms. These results highlight the cross-platform consistency and reproducibility of key protein associations across NDs.

Once we identified proteins associated with each disease, we determined which proteins were associated with multiple NDs. SMOC1 (*P*_AD_ = 7.4 × 10^−6^, *P*_PD_ = 1.7 × 10^−7^, *P*_FTD_ = 3.4 × 10^−3^), previously reported associated with AD^8,19^, was associated with all NDs. Other commonly dysregulated proteins included NPTXR (*P*_AD_ = 4.9 × 10^−136^, *P*_PD_ = 8.5 × 10^−19^, *P*_FTD_ = 5.8 × 10^−25^), which exhibited consistently decreased protein levels across all NDs, indicating a shared disruption in synaptic integrity. Other examples include UCHL1 (*P*_AD_ = 6.5 × 10^−7^, *P*_PD_ = 2.3 × 10^−23^, *P*_FTD_ = 8.1 × 10^−4^), a proteasomal enzyme associated with neuronal injury^25^; the immune modulator kinase MAPK1 (*P*_AD_ = 1.4 × 10^−18^, *P*_PD_ = 5.1 × 10^−5^, *P*_FTD_ = 1.1 × 10^−2^)^26^; and GFAP (*P*_AD_ = 3.3 × 10^−6^, *P*_PD_ = 2.9 × 10^−2^), indicative of astrocytic activation^27^.

**Fig. 3:**
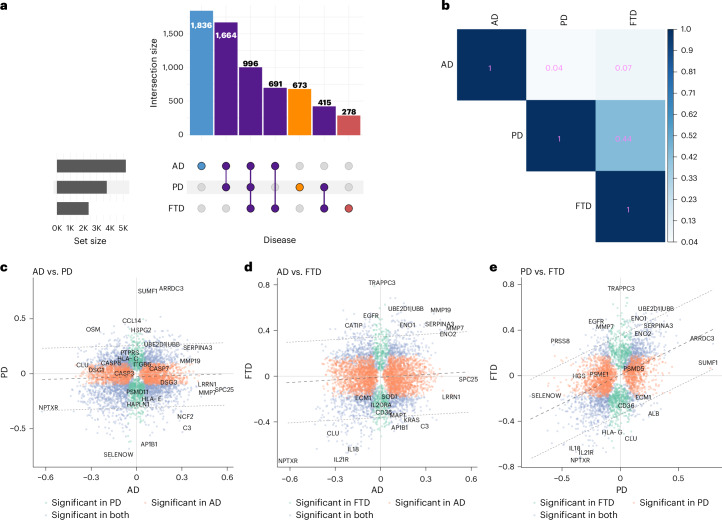
Overlap and effect size correlation of proteins associated with AD, PD and FTD. **a**, An upset plot visualizing the overlap between proteins associated with each ND. **b**, Pairwise correlation matrix of effect sizes for differentially abundant proteins across AD, PD and FTD. The color gradient represents the strength of correlation from low (white) to high (dark blue). **c**–**e**, Scatter plots display pairwise comparisons of effect sizes for proteins in AD versus PD (**c**), AD versus FTD (**d**) and PD versus FTD (**e**). Each point represents a protein, with the *x* axis and *y* axis representing the effect sizes in the respective diseases. Proteins significantly associated with both diseases are shown in blue; proteins uniquely significant in the first and second diseases are represented in orange and teal colors, respectively. The dark gray dashed line in the middle represents the regression line; the light gray outer dashed lines represent the 95% confidence interval bounds. Key proteins with notable effect size changes are labeled with the protein name.

A total of 996 proteins (15.2%) were associated with AD, PD and FTD, suggesting the presence of shared pathological mechanisms (Fig. 3a and Extended Data Fig. 1b). Additionally, 1,664 proteins (25.4%) were shared between AD and PD. Among those, 286 (17%) were consistently increased; 483 (29%) were consistently decreased; and 895 (54%) had opposite direction, between AD and PD. Fewer overlaps were observed between AD and FTD (691 proteins, 10.5%) and between PD and FTD (415 proteins, 6.33%), reflecting their unique molecular characteristics. Notably, AD exhibited the largest number of disease-specific proteins (1,836, 28%), followed by PD (673, 10.3%) and FTD (278, 4.24%).

### Assessment of shared and disease-specific molecular changes across neurodegeneration

To further investigate the degree of proteomic similarity across AD, PD and FTD, we performed pairwise correlation analyses of effect sizes for all the proteins associated with NDs. Although the correlation between AD and PD was minimal (*r*^2^ = 0.04), higher correlation was observed between PD and FTD (*r*^2^ = 0.44), indicating substantial overlap in proteomic changes between these two NDs. The correlation between AD and FTD remained low (*r*^2^ = 0.07), reflecting the distinctive molecular features of these disorders (Fig. 3b).

To identify the key proteins driving these associations, we analyzed those with consistent effect sizes as well as those falling outside the 95% confidence interval for effect size correlation (Fig. 3c–e and Supplementary Table 4). The absence of a strong correlation between AD and PD highlighted the divergent proteomic landscapes of these diseases. Proteins with large effect sizes were often disease specific or beyond the 95% confidence interval for effect size correlation. Some proteins were associated with both diseases but showed opposite directions of effect, suggesting their potential utility in differential diagnosis. The examples include SPC25 (*β*_AD_ = 0.58, *P*_*AD*_ = 2.1 × 10^−102^; *β*_PD_ = −0.13, *P*_PD_ = 2.7 × 10^−3^) and LRRN1 (*β*_AD_ = 0.47, *P*_AD_ = 1.1 × 10^−71^; *β*_PD_ = −0.1, *P*_PD_ = 6.2 × 10^−2^), among others. In addition, there are proteins that were associated with both diseases but exhibited substantially higher effect sizes in one disease compared to the other: SUMF1 (*β*_PD_ = 0.77, *P*_PD_ = 2.3 × 10^−54^; *β*_AD_ = 0.08, *P*_AD_ = 3.1 × 10^−3^) and ARRDC3 (*β*_PD_ = 0.80, *P*_PD_ = 8.9 × 10^−82^; *β*_AD_ = 0.22, *P*_AD_ = 9.1 × 10^−22^). Proteins central to mitochondrial dysfunction and proteasomal degradation, including PSMC5 (*β*_PD_ = 0.36, *P*_PD_ = 1.4 × 10^−56^; *β*_AD_ = 0.04, *P*_AD_ = 4.1 × 10^−3^) and HGS (*β*_PD_ = −0.35, *P*_PD_ = 2.1 × 10^−118^; *β*_AD_ = −0.09, *P*_AD_ = 4.5 × 10^−24^), also showed higher effect size in PD.

AD and FTD (*r*^2^ = 0.07) also exhibited limited proteomic overlap. This difference was driven by several signal transduction-related proteins, including KRAS, AP1B1 and EGFR, which displayed at least a twofold difference in effect size between AD and FTD. Despite the overall weak correlation, a subset of proteins displayed consistent direction and similar effect sizes in both diseases, including proteins involved in glycolysis, such as ENO2, and synaptic dysfunction markers such as NPTXR and CLU.

The relatively higher correlation observed between PD and FTD (*r*^2^ = 0.44) suggests a degree of shared pathophysiology, potentially linked to protein degradation and synaptic dysfunction. In particular, proteins involved in the ubiquitin–proteasome system, such as UBE2D1 | UBB and UBE2D3 | UBB, were significantly elevated in both diseases. Moreover, the concurrent elevation of inflammatory mediators such as TRAPPC3 and the shared reduction in interleukin proteins (IL-18 and IL-21R) suggest converging mechanisms of neuroinflammation across both diseases. Notably, synaptic proteins, such as CLU (*β*_PD_ = 0.1, *P*_PD_ = 1.1 × 10^−2^; *β*_FTD_ = −0.53, *P*_FTD_ = 2.3 × 10^−14^) and MMP7 (*β*_PD_ = −0.1, *P*_PD_ = 1.1 × 10^−2^; *β*_FTD_ = 0.39, *P*_FTD_ = 5.8 × 10^−9^), exhibit markedly divergent patterns with opposite effect size direction across both diseases.

Overall, these findings suggest that, although each disorder exhibits distinct proteomic changes, they also share converging mechanisms involving protein clearance deficits and synaptic dysfunction.

### Differential pathway dysregulation across AD, PD and FTD

**Fig. 4:**
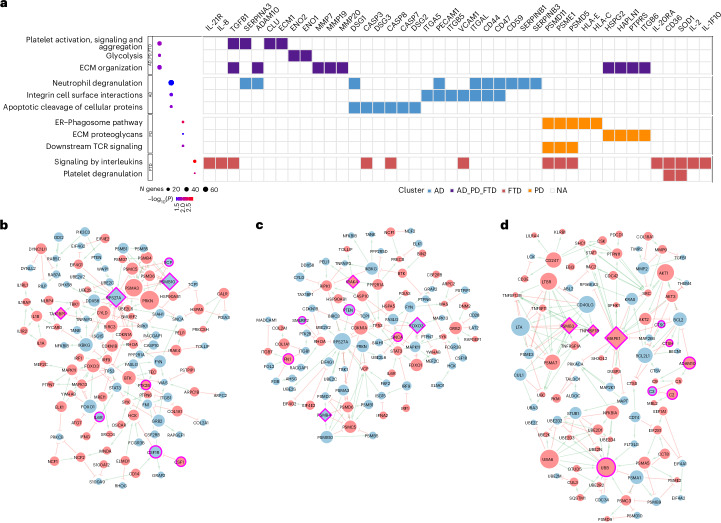
Pathway enrichment and network analyses for AD, PD and FTD. **a**, The dot plot displays selected pathways across NDs enriched in proteins overlapping across AD, PD and FTD as well as those proteins unique to each disease. Pathway clusters are indicated by the labels on the left. Dot size represents the number of identified genes, and the color gradient reflects FDR-adjusted significance. The tile plot on the right side highlights differentially expressed proteins within each pathway, with color coding corresponding to the associated disease. **b**–**d**, Graph-based representations of the disease-specific PPI networks for AD (**b**), PD (**c**) and FTD (**d**). In each network, green edges indicate activating interactions, and red edges represent inhibitory interactions. Node color reflects the direction of disease-associated change: proteins with increased expression levels in disease are shown in red, and those having decreased expression levels are shown in blue. Key upstream regulators, predicted through network perturbation analysis, are depicted as diamond-shaped nodes; all other proteins are shown as circles, with node size representing the out-degree (regulatory edges going out of the node). Both key upstream regulators and other important proteins in the network are highlighted by a pink boundary. NA, not applicable.

To determine how the common and disease-specific proteins point to biological mechanisms implicated in disease, we performed pathway enrichment analysis on differentially abundant plasma proteins across AD, PD and FTD (Fig. 4a and Supplementary Table 5).

**Extended Data Fig. 5:**
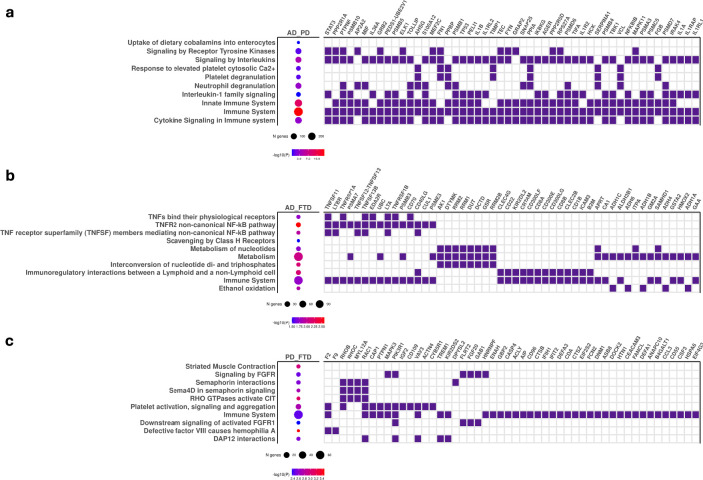
Pathway enrichment analysis of proteins commonly associated with multiple neurodegenerative disorders. (Left) The dot plot displays top 10 pathways enriched in proteins overlapping across multiple disease. Pathway clusters are indicated by the labels on the left. Dot size represents the number of proteins associated with a particular pathway, and the color gradient reflects significance of pathway. (Right) The tile plot highlights differentially expressed proteins within each pathway. **a**, We identified 44 Reactome pathways to be significantly (FDR < 0.05) enriched in 1,664 proteins commonly associated with both AD and PD. **b**, 10 pathways revealed significant enrichment (FDR < 0.05) in 691 AD- and FTD-associated proteins. **c**, A total of 115 pathways showed nominal enrichment (*p* < 0.05) with 415 protein commonly shared between PD and FTD.

Disease-specific proteins, defined as significant in one and not in the others (AD = 1,836; PD = 673; FTD = 278), were associated with 164, 136 and 83 pathways, respectively (Extended Data Fig. 1b). Although some pathways were uniquely associated with a single disease, others overlapped across multiple NDs. The pathway enrichment analyses for proteins overlapping across different diseases (AD–PD–FTD = 996, AD–PD = 1,644, AD–FTD = 691 and PD–FTD = 415) identified 101 pathways associated with proteins commonly identified in all three NDs, 44 for AD and PD, 10 for AD and FTD and 115 for PD and FTD (Extended Data Fig. 5).

Pathways that were significantly enriched across all three diseases include ‘Platelet activation, signaling and aggregation’ (*P* = 4.2 × 10^−4^), ‘Glycolysis’ (*P* = 5.0 × 10^−4^), ‘Immune system’ (*P* = 2.0 × 10^−12^) and ‘Extracellular matrix (ECM) organization’ (*P* = 6.1 × 10^−4^). Dysregulated ECM proteins, including matrix metalloproteinase family members such as MMP7 (*β* > −0.10, *P* < 1.1 × 10^−2^), MMP19 (*β* > 0.14, *P* < 1.7 × 10^−3^) and MMP20 (*β* > −0.17, *P* < 1.2 × 10^−2^), are mostly produced by microglia or macrophages^28^ and are consistent with previous studies implicating ECM remodeling in neurodegeneration^29^. Proteins in this pathway were enriched in human microglia and macrophages (*P* = 5.7 × 10^−3^). Similarly, glycolysis emerged as a commonly enriched pathway across AD, PD and FTD, involving key proteins such as enolases (ENO1 and ENO2). Enolases, particularly ENO2, a neuron-specific isoform, have been implicated in NDs due to their role in energy metabolism and neuronal survival^30^. Finally, the immune system emerged as a super pathway compromised across all NDs, including multiple ubiquitin (UBE2C, UBE2L6 and RPS27A), proteasome (PSMB3 and PSMB10), kinase (MAPK1, MAPK11, MAPK13 and IRAK4) and cathepsin (CTSC, CTSH and CTSV) family proteins, among others.

Proteins uniquely associated with AD were enriched in ‘Apoptotic cleavage of cellular proteins’ (*P* = 5.5 × 10^−5^; CASP3, CASP7 and CASP8), ‘Integrin cell surface interactions’ (*P* = 9.7 × 10^−5^; ITGA5, ITGB5 and ITGAL) and ‘Neutrophil degranulation’ (*P* = 1.9 × 10^−4^; C2, C3, CD44, CD47 and CD59) pathways. Furthermore, integrins such as ITGA5, ITGB5 and VCAM1, mainly expressed in endothelial cells (*P* = 6.9 × 10^−8^), were part of the ‘Immune system’ (*P* = 1.3 × 10^11^) and integrin cell surface interaction pathways and likely reflect disruptions in cell adhesion and blood–brain barrier integrity, consistent with reports of vascular dysfunction in AD^31^.

Pathways enriched in PD-specific proteins were predominantly linked to the ‘ER-Phagosome pathway’ (endoplasmic reticulum–phagosome; *P* = 2.9 × 10^−3^) and ‘ECM proteoglycans’ (*P* = 0.04), involving HLA proteins (HLA-C and HLA-E) and proteasomes (PSME1, PSMD5, PSMB10 and PSMD11). In proteins uniquely associated with FTD, we identified significant enrichment of pathways associated with ‘Signaling by interleukins’ (*P* = 1.1 × 10^−3^) and ‘Platelet degranulation’ (*P* = 6.4 × 10^−3^), characterized by the proteomic alterations in cytokine receptors (IL-2, IL-18, IL-1F10 and IL-21R). Notably, dysregulated platelet activity in FTD, supported by altered expression of SOD1 and CD36, may indicate a previously underappreciated role of vascular contributions to FTD pathogenesis^32^.

To conclude, the pathway enrichment analysis reveals a complex interplay of shared and disease-specific molecular mechanisms across AD, PD and FTD, with converging pathways suggesting common immune (CLU and TGFB1), glycolytic (ENO1 and ENO2) and matrisome (ADAM10 and MMP7) disruptions, whereas distinct proteomic signatures underscore disease-specific pathophysiological processes such as apoptosis (CASP3 and CASP7) in AD, ER-Phagosome (PSME1 and PSMD5) impairment in PD and platelet dysregulation (SOD1 and CD36) in FTD. Mapping these enriched pathways to human cell types, including microglia/macrophages, astrocytes and endothelial cells, provides a refined understanding of the cellular context driving neurodegenerative processes, underscoring both shared vulnerabilities and unique pathological hallmarks.

### Network analysis of upstream regulators and mechanistic hubs

**Extended Data Fig. 6:**
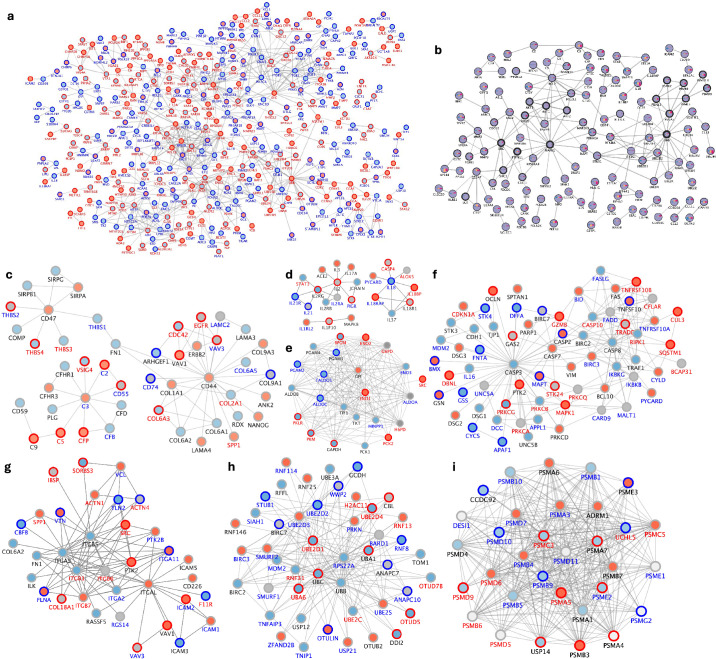
Protein-protein interaction networks. **a**, Proteins commonly associated with AD, PD, and FTD. The network shows up (red) and down (blue) regulation of commonly associated proteins in AD, PD, and FTD with FDR < 0.05. Colors of the node (AD), label (PD), and node border (FTD) reflect the dysregulation direction, i.e. upregulation and downregulation, for each disease. **b**, Commonly associated proteins that are enriched in pathways. The network consists of proteins significantly associated with the disease (FDR < 0.05) that overlap across AD, PD, and FTD. The width of node borders reflects the number of pathways enriched by each protein. The bar graphs inside each protein node depict relative effect sizes of AD (blue), PD (orange), and FTD (pink). **c-i**, Significantly disease-associated proteins that are involved in known protein families. Subnetwork of proteins from **c**, complement system, **d**, interleukin, **e**, enolase, **f**, caspase, **g**, integrin, **h**, ubiquitin, and **i**, proteasome families. Colors of the node (AD), label (PD), and node border (FTD) reflect the dysregulation direction, i.e. upregulation (red) and downregulation (blue), for each disease. Grey/black colors imply absence of significant (FDR < 0.05) association between the disease and the protein. Nodes that are part of the protein family and not significantly associated with a disease of interest are shown in green, where applicable.

Understanding protein interactions is essential for deciphering their coordinated roles in maintaining biological processes. To investigate how dysregulation in protein expression can propagate through functional networks and affect these processes, we constructed protein–protein interaction (PPI) networks using proteins associated with AD, PD and FTD (Extended Data Fig. 6c–i). We applied a network modeling approach that infers directionality. This approach enabled us to infer potential upstream regulators and identify proteins whose expression perturbations are predicted to produce maximal downstream effects within each disease-specific network. These upstream regulators can modulate the expression of downstream targets through direct or indirect (via mediator proteins) interactions (Fig. 4b–d).

The AD-specific network comprised 114 nodes and 196 directed interactions (Fig. 4b), centering around proteins associated with immune regulation, cytoskeletal remodeling and proteostasis pathways. Network perturbation analysis identified PSMB10, RPS27A and TAX1BP1 as key upstream regulators predicted to influence the expression state of multiple downstream proteins (Supplementary Table 6). VCP, a potential therapeutic target for AD^33^, was identified as a downstream target of PSMB10 (via indirect interaction through PSMD6), a proteasomal subunit known for its essential role in protein degradation and immune response. Other downstream targets, including the immune-related proteins IL-6R and CSF1R, both associated with microglial activation, showed regulatory connections with PSMB10 and TAX1BP1. PTK2B, another important node and an established AD genome-wide association study (GWAS) locus^34^, was regulated by CASP10, a caspase involved in apoptotic signaling^35^.

The PD network featured 95 nodes and 133 interactions (Fig. 4c). IRAK4, a central kinase in TLR/IL-1 signaling^36^, was identified as a key upstream regulator and directly connected to PPP2R1A, a serine/threonine phosphatase implicated in neurodegeneration^37^, as well as RIPK1, a kinase involved in cell death and inflammation that is elevated in the brains of PD mouse models^38^. Another upstream regulator, FOXO3, was linked to stress response proteins including MAPK11, MAPK13, STAT3 and YWHAB, placing it at the intersection of apoptosis, immune response, oxidative stress and metabolic adaptation^39^. SNCA, the core protein in Lewy body pathology, was connected to PTEN and SMURF2 through shared partners in the proteostasis subnetwork. PTEN, a tumor suppressor that negatively regulates PI3K/AKT signaling^40^, was regulated by FOXO3 and connected to several ubiquitin-related proteins (for example, UBE2L6 and UBE2C). These links position PTEN as a central signaling hub influencing both neuronal resilience and degenerative processes in PD^41^.

The FTD-specific network consisted of 94 nodes and 191 interactions (Fig. 4d), with prominent involvement of lysosomal hydrolases, complement cascade proteins and MAPK signaling components. The upstream regulator MAPK1, a canonical ERK kinase, directly controlled the expression of MAPT, PRKACA and PTPN11. Notably, in silico perturbation of MAPK1 alone influenced the expression levels of 29 other proteins, accounting for nearly one-third of the network, emphasizing its broad regulatory impact (Supplementary Table 6). MAPT, a hallmark of tauopathies, was centrally positioned in this network, forming directional links with kinase regulators and phosphatase-associated modules. CTSC and CTSH, lysosomal cysteine proteases essential for proteolytic degradation, appeared downstream of complement proteins C2 and C3 via Bcl-2 family members. ADAM10, a protease involved in synaptic remodeling and Notch signaling^42^, was integrated within this network and regulated MAPK1, highlighting a link between inflammatory signaling and synaptic dysfunction in FTD^43^.

In summary, disease-specific network modeling uncovered key regulatory proteins, such as RPS27A in AD, IRAK4 in PD and MAPK1 in FTD, that orchestrate widespread molecular changes across NDs. These findings highlight the converging roles of ubiquitination and kinase-associated proteins in regulating proteostasis, immune signaling and stress response pathways in the pathogenesis of AD, PD, and FTD.

### Disease-specific biomarker panels

**Fig. 5:**
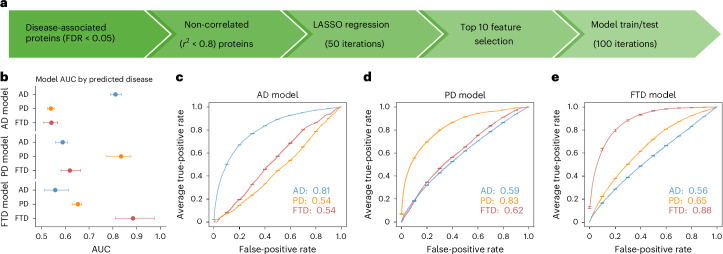
Disease-specific predictive models for AD, PD and FTD. **a**, The pipeline for identifying disease-specific biomarker panels. **b**, AUC values with 95% confidence intervals from the testing dataset for each disease-specific model tested against all three ND groups: AD (*n* = 1,162), PD (*n* = 314) and FTD (*n* = 98). Each row corresponds to a prediction model trained for one disease and applied to all disease groups (color coded: AD in blue, PD in orange and FTD in pink). The bars and error bars represent bootstrapped (*n* = 100) means and 95% confidence intervals. **c**–**e**, ROC curves showing average performance of the AD (**c**), PD (**d**) and FTD (**e**) models in distinguishing their respective target disease (same color line as the legend) from the remaining groups.

To create disease-specific plasma predictive models, we used least absolute shrinkage and selection operator (LASSO) regression, trained iteratively (*n* = 100) using 70% of the dataset and tested in 30% of the data, with class balancing to ensure equal representation of cases and controls during training and testing (Fig. 5a). To examine the specificity of each biomarker panel, trained models were also tested on other NDs not included during model training (see Methods for details).

These plasma-based prediction models demonstrated strong performance in distinguishing each ND from cognitively normal controls. The models achieved area under the receiver operating characteristic (ROC) curve values of 0.81 for AD (95% confidence interval: 0.79–0.84), 0.83 for PD (95% confidence interval: 0.77–0.88) and 0.88 for FTD (95% confidence interval: 0.81–0.97) (Fig. 5b,e and Supplementary Table 7). When applied to other NDs, each model demonstrated reduced performance, highlighting their disease specificity. For example, the AD panel yielded near-random classification accuracy in PD (area under the curve (AUC) = 0.54) and FTD (AUC = 0.54), whereas the FTD panel showed limited predictive power for AD (AUC = 0.56) and PD (AUC = 0.65). The poor cross-disease performance of the AD panel suggests a high degree of biomarker specificity and limited overlap in proteomic alterations with PD and FTD, consistent with our across-disease effect size correlation results. In contrast, the slightly better performance of the FTD panel in PD aligns with shared pathological features and more consistent proteomic changes between these two diseases, as evidenced by a higher correlation in protein effect sizes (*r*^2^ = 0.44).

**Extended Data Fig. 7:**
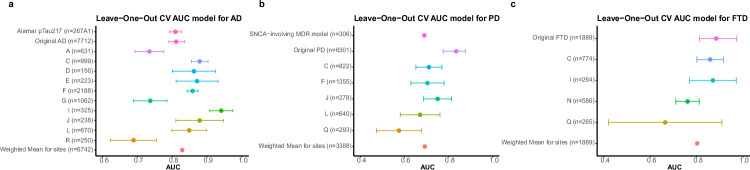
Leave-One-Site-Out Cross-Validation (CV) of Disease-Specific Prediction Models for AD, PD, and FTD. We applied LASSO logistic regression with leave-one-site-out cross validation (LOOCV), which includes 6,742 participants in AD model, 3,388 participants for PD model, and 1,889 participants for FTD model. **a–c**, Area under the receiver operating characteristic curve (AUC) with 95% confidence intervals (CIs) for models predicting **a**, Alzheimer’s disease (AD), **b**, Parkinson’s disease (PD), and **c**, frontotemporal dementia (FTD). Each point represents model performance on a single held-out site. The ‘Original’ model in each panel refers to performance on the full dataset without site-level CV (Fig. 5 in main manuscript). In A and B, top rows show AUCs from previously published reference models. Site-specific rows correspond to leave-one-site-out CV results, in which data from one site is held out for testing while the model is trained on all others. The red dot indicates the weighted mean AUC across all sites, weighted by sample size. For the AD cohort, site-specific Area Under the Curve (AUC) values ranged from 0.689 to 0.941. The sample size-weighted mean AUC was 0.829, indicating strong discrimination between AD and control samples across independent sites. The original model, trained using data from all sites, achieved an AUC of 0.811. Additionally, we included a model utilizing ptau-217 to predict AD using internal dataset, the largest site (site F), which yielded an AUC of 0.809 as a reference. Overall, these results demonstrate robust performance in distinguishing AD and the initial model. The PD models resulted in a slightly lower overall mean AUC of 0.692, with the original model achieving a higher AUC of 0.835. This suggests that while the PD models are generally effective, there is variability in discrimination performance across different sites. For FTD, the sample size-weighted mean AUC across contributors was 0.802, indicating stable performance across sites. The original FTD model showed an impressive AUC of 0.884. These findings highlight the model’s reliability and strong predictive capability for FTD across different contributors.

Notably, we also compared the prediction model AUCs with that of plasma ptau217 for AD and synuclein seeding for PD (Extended Data Fig. 7). Our models showed similar discriminatory power as of these well-established biomarkers. This is important as new treatments targeting tau, Aβ or synuclein are being implemented; therefore, new biomarkers independent of these proteins need to be developed. In summary, these findings highlight the consistent predictive accuracy of disease-specific biomarker panels for AD, PD and FTD in distinguishing them from healthy controls while also reflecting differences in cross-disease applicability based on the extent of molecular overlap and divergence.

## Discussion

This large-scale plasma proteomics analysis provides a comprehensive molecular profile of AD, PD and FTD, highlighting both shared and disease-specific alterations (Fig. 2 and Supplementary Tables 1 and 4). Among the commonly dysregulated proteins, SMOC1, previously reported to be associated with AD^44,45^, was significantly elevated across all three diseases. Its effect sizes in AD and PD were similar and passed FDR significance, indicating a broader role in neurodegeneration beyond AD. SMOC1 has been implicated in ECM remodeling^46^ and co-localizes with amyloid plaques and tau pathology in human brains^7^, supporting its involvement in neurodegenerative processes. Its consistent upregulation across diseases highlights its potential as a pan-neurodegenerative biomarker. Pathway enrichment analysis of proteins shared across AD, PD and FTD further supports this finding, with ‘ECM organization’ emerging as a significantly enriched pathway that includes several matrix metalloproteinases (MMP7, MMP19 and MMP20). Additionally, the consistent downregulation of NPTXR, a synaptic protein, aligns with growing evidence that synaptic dysfunction is a common pathological feature across NDs^22^. Another protein consistently associated with all diseases was MAPK1, which, along with other kinases such as MAPK11, MAPK13 and IRAK4, was involved in immune system pathway disruption in AD, PD and FTD. These findings are consistent with previous studies implicating mitogen-activated protein (MAP) kinase signaling in innate immune regulation and suggesting its potential as a therapeutic target in neurodegeneration^26,47^.

AD exhibited the most extensive disease-specific alterations, followed by PD and FTD, reflecting the heterogeneous nature of these disorders (Fig. 3a). In AD, the upregulation of multiple apoptotic regulators, including CASP3, CASP7 and CASP8, supports a central role for programmed cell death in neuronal loss^48^, which was one of the most significant AD-specific pathways (‘Apoptotic cleavage of cellular proteins’). Caspases such as CASP3 and CASP8, known regulators of apoptosis^35,49^, displayed opposing effect size directions in AD and PD (Fig. 3c), highlighting their potential as differential biomarkers. CASP10, another apoptosis-associated caspase^35^, was identified in the AD-specific network as an upstream regulator of PTK2B, an established AD GWAS locus^34^, reinforcing the link between caspase-mediated protein turnover and AD pathophysiology^49^. Enrichment of AD-associated proteins in endothelial-related and microglial/macrophage-related markers points to vascular dysfunction and neuroinflammation as key contributors to disease progression^50,51^. These regulators collectively modulated key downstream proteins involved in AD, including VCP, a known therapeutic target for AD^33,52^, as well as central immune mediators such as IL-6R and CSF1R^45,53^. Together, these findings suggest that impaired protein degradation and chronic immune activation are tightly interlinked features of the AD plasma proteome^45,53,54^.

PD was marked by prominent dysregulation of ubiquitin pathway components, including HGS, ARRDC3, UBE2L6 and UBE2C, consistent with the well-established role of impaired protein degradation in PD pathogenesis^55,56^. Ubiquitin-related proteins were also significantly altered in FTD, contributing to the notable molecular similarity between PD and FTD (*r*^2^ = 0.44; Fig. 3b). For example, UBE2D1 | UBB and UBE2D3 | UBB were elevated in both disorders. These results align with enrichment of these PD-associated proteins in pathways such as ‘ER-Phagosome’ and ‘ECM proteoglycans’, which are implicated in ubiquitination defects and the accumulation of misfolded proteins, including α-synuclein^57^. Key upstream regulators identified in the PD-specific protein interaction network included IRAK4 and FOXO3, known to modulate inflammatory and oxidative stress responses^36,39^. Through PTEN and SNCA, these regulators controlled a cascade of downstream ubiquitin proteins in the network, reinforcing their critical role in maintaining cellular stress response and α-synuclein clearance^36,41^.

FTD-specific associated proteins were associated with ‘Interleukin signaling’ and ‘Platelet degranulation’ pathways, pointing to cytokine imbalance and possible vascular contributions to disease pathology^32^. Key proteins driving these associations included members of the interleukin family (IL-20RA, IL-2 and IL-1F10) and lysosomal proteases (CTSC, CTSH and CTSV). Notably, CTSC and CTSH, consistently dysregulated across AD, PD and FTD in our analysis, were previously identified as potential causal proteins in AD, supported by proteome-wide association study, co-localization analyses and Mendelian randomization. These proteases, along with ADAM10, were closely connected to complement cascade components (C2 and C3) and MAPK signaling proteins (such as MAPK1), reflecting a network architecture aligned with known roles of inflammation, lysosomal dysfunction and synaptic remodeling in FTD^58^. MAPK1 emerged as a central regulatory node, influencing the expression of nearly one-third of proteins in the FTD-specific network and directly modulating MAPT and complement system proteins (Fig. 4d), emphasizing the importance of kinase dysregulation in tauopathy progression^59,60^.

The disease-specific biomarker panels demonstrated consistent predictive performance across all NDs (Fig. 5 and Supplementary Table 7). Notably, the developed models showed high disease specificity. The poor cross-disease performance of the AD panel is consistent with its unique proteomic alterations involving immune response^6,61^, lipid metabolism^6^ and amyloid-related processes^62^. The modest cross-applicability of the FTD panel to PD supports the high proteomic overlap (*r*^2^ = 0.44) as well as shared clinical and pathological features between these diseases^63^.

Although our study leverages large-scale cross-sectional plasma proteomic datasets across NDs, several limitations should be considered when interpreting the findings. First, lower sample sizes for FTD and PD may limit statistical power and the ability to detect disease-specific alterations with high confidence. This is particularly important for FTD, which encompasses clinically and biologically heterogeneous subtypes that likely have distinct proteomic signatures^64^. Another limitation of this study is the limited availability of race and ethnicity data, with more than 60% of the GNPC data lacking this information at the time of analyses. Among participants with available data, most were identified as White, which may limit the generalizability of our findings across diverse populations. Second, although the SomaScan platform provides broad proteome coverage^65^, we could not validate most of our findings using orthogonal technologies (for example, Olink, mass spectrometry or Alamar data) due to the limited availability of sufficiently large datasets, which is critical to ensure cross-platform reproducibility and technical reliability^66^. Also, an intrinsic limitation of the SomaScan platform is that phosphorylated tau isoforms such as p-tau181 and p-tau217, which are well-established plasma biomarkers for AD diagnosis and progression, are not captured by the current SomaScan panel. We compared the predictive models generated in this study with plasma p-tau217, which is available for Site F (the Knight ADRC cohort), which is the largest contributor of GNPC version 1, and with the α-synuclein seeding assays, showing that the SomaLogic-based models are as good as p-tau217 and α-synuclein seeding assays. It is important to develop new predictive models that are not capturing only previously known pathology, as current therapies are targeting those proteins. Individuals being treated with those therapies could become biomarker negative even though they are not cured and, in fact, still show disease progression. Although biomarker-based classification is advancing, particularly in AD due to the availability of amyloid and tau positron emission tomography imaging, clinical diagnosis remains central for diseases such as PD and FTD, where such imaging tools are lacking. This reliance on clinical assessments may introduce some diagnostic misclassification, potentially reducing power and underestimating the extent of molecular overlap across NDs^67^. Lastly, the lack of patient medication data limits our ability to account for treatment-related proteomic changes. As emerging therapies increasingly target disease mechanisms, future studies incorporating detailed treatment information and focusing on biomarker changes independent of amyloid or tau are crucial for tracking therapeutic response and disease progression^68^.

To conclude, our large-scale plasma proteomics study offers a comprehensive molecular dissection of AD, PD and FTD. By identifying distinct and overlapping protein signatures, developing accurate disease-specific prediction models and uncovering converging and divergent biological pathways, this work enhances understanding of ND heterogeneity and supports the utility of plasma-based biomarkers for advancing non-invasive diagnostics and precision medicine approaches.

## Methods

### Study participants, plasma samples and proteomics profiling

GNPC version 1 consists of 23 independent sites contributing plasma proteomic samples from individuals with a range of clinical backgrounds, including AD, PD, FTD, amyotrophic lateral sclerosis, diabetes, cancer and cognitively normal controls. Ethics approval for each individual site was obtained from its respective institutional review board (IRB), and the research was conducted following the approved protocols (Washington University in St. Louis (WUSTL) IRB approval no. 201109148). Written informed consent was obtained from all participants or their family members, and the study design was approved by all participating institutions. For this study, we focused on participants diagnosed with three major NDs (AD, PD and FTD) as well as healthy controls. A total of 10,527 cross-sectional plasma samples (AD = 1,936, dementia = 1,638, PD = 525, FTD = 163 and controls = 6,265) from 16 independent contributor sites were analyzed.

For both PD and FTD groups, detailed staging information (such as Hoehn and Yahr for PD or clinical severity scores for FTD) was not available. Moreover, subtyping of FTD cases by underlying pathology (for example, tau, TDP-43 or FUS) was not performed due to lack of corresponding pathological or genetic confirmation. As such, our FTD group reflects a clinically defined, binary classification (that is, presence or absence of disease). While AD includes cases at different stages, 80% of the participants had a CDR = 1, which corresponds to early disease stages. PD primarily includes early-stage cases without formal dementia (that is, prior to Parkinson’s disease dementia). Individuals categorized as dementia based on clinical tests but without confirmed AD diagnosis were not included in the main analyses, although additional sensitivity analyses were performed including participants with AD and those with dementia. For individuals with longitudinal proteomic measurements, only the most recent timepoint was used.

Plasma samples were generally collected via blood draw in the morning or midday, typically without requiring participants to fast. All samples underwent standardized preparation and processing protocols and were stored at −80 °C until proteomic profiling.

Proteomic analysis was conducted using the SomaScan assay version 4.1 (SomaLogic), a highly sensitive, aptamer-based platform. Although sample preparation was conducted separately at each site^69^, all samples were subsequently sent to SomaLogic for centralized protein quantification using the SomaScan platform. The samples were not processed in replicates, but non-sample calibrators, which are used in the SomaScan assay to assess the variability in the proteomic measurements, were run in triplicates (three per plate). This assay uses chemically modified nucleotide sequences (aptamers) to convert protein abundance into a nucleotide signal, which is quantified using fluorescence on microarrays^70^. SomaScan version 4.1 measures 7,584 aptamers, targeting approximately 6,400 unique human proteins. The proteins targeted by this platform cover a broad spectrum of biological processes, including inflammation, cancer and cardiovascular function, and it detects secreted, intracellular and extracellular proteins, including receptors, kinases, growth factors and hormones^71^. The assay has been extensively validated in human plasma and serum^70^, demonstrating high reproducibility and reliability. Previous studies reported median coefficients of variation around 5% for both intra-plate and inter-plate assessments^65,70,72^. With a detection range spanning from femtomolar to micromolar concentrations, the SomaScan platform offers high sensitivity, surpassing that of conventional immunoassays. The SomaScan version 4.1 platform used in this study does not include aptamers specific to different Aβ and phosphorylated tau isoforms (for example, Aβ42 or p-tau217), and, therefore, these biomarkers were not measured.

### Quality control and normalization of proteomic data

The proteomic dataset from SomaLogic provided quantitative measurements of protein aptamers in relative fluorescence units (RFU). Measurements were available for 7,584 aptamers mapping to approximately 7,000 unique proteins. Initial data normalization was performed by SomaLogic to remove any technical inter-plate and intra-plate variability^73^.

**Extended Data Fig. 8:**
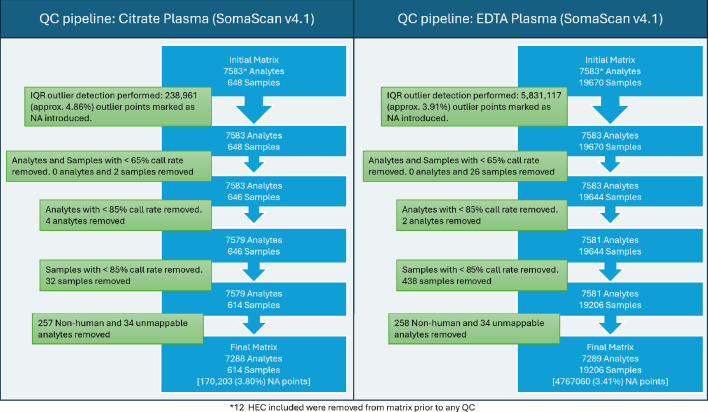
Proteomics data quality control (QC) check pipeline. The QC pipeline was applied separately to both citrate and Ethylenediaminetetraacetic acid (EDTA) plasma samples. The two main filters include interquartile range (IQR) outlier detection and call rate. Aptamers with a median coefficient of variation (CV) greater than 0.15 or those with values falling outside 1.5 times the IQR in more than 85% of the samples were removed. After implementing all QC steps, a total of 10,527 samples and 7,289 aptamers passed the quality assessment.

Further quality control was implemented using an internally developed protocol at both the aptamer and sample levels^74,75^. First, we applied log_10_ transformation to the RFU proteomic values to approximate a normal distribution. Outliers were then identified using an interquartile range (IQR)-based statistical approach. Any proteomic measure that was lower than Q1 − 1.5× IQR and higher than Q3 + 1.5× IQR, where Q1 and Q3 are the first and third quartiles, respectively, was marked as an outlier and set to missing (Extended Data Fig. 8). A 65% call rate threshold was then applied such that any sample or analyte that had a call rate lower than 65% was removed from the matrix. This was followed by recalculation of call rate and second pass removal of analytes and samples using a stringent 85% call rate. Finally, analytes targeting non-human proteins or those missing proper annotations were removed. Data from all contributing sites underwent a unified quality control process. However, plasma samples that used EDTA as anticoagulants were processed separately from those that used sodium citrate (Site E). At the end of the quality control process, a total of 10,527 samples and 7,289 aptamers were retained.

**Extended Data Fig. 9:**
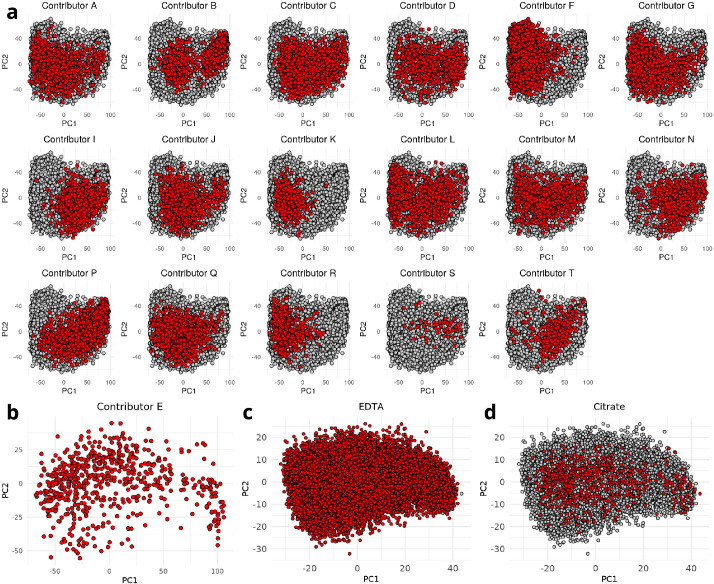
Principal component analysis (PCA) of proteomic data before and after z-score based normalization. **a**, The PCA plot of ethylenediaminetetraacetic acid (EDTA) plasma samples from each contributor in GNPC version 1. The red dots represent first two proteomic principal components (PCs) of samples from an individual contributor against the grey dot representing proteomic PCs of all sample from every contributor. **b**, The PCA plot of sodium citrate plasma samples from a single contributor ‘K’. **c-d**, The PCA plots of z-score normalized proteomic data from the **c**, EDTA and **d**, Citrate samples across all contributors.

To identify any potential batch effects, principal component analysis plots were generated for visualization (Extended Data Fig. 9). Missing values within the dataset were imputed using a bootstrapping approach. Finally, aptamer levels in the EDTA and citrate plasma samples were *z*-score normalized by applying a log_10_ transformation followed by normalization using the ‘scale’ function in R, with both the scale and center options set to TRUE. *z*-score normalization was performed separately for citrate samples from a single contributor and for the pooled EDTA samples from all other contributors.

### Cognitive assessments

Participants from each contributing site underwent comprehensive phenotyping, which included longitudinal evaluations and, where available, standardized cognitive, neurological and neuropsychological assessments. Board-certified neurologists and neuropsychologists conducted these evaluations using established cognitive scales, such as the global CDR^17^ and the MMSE^18^, where applicable. When applicable, dementia diagnoses were assigned based on criteria established by the National Institute of Neurological Disorders and Stroke^76^ and the National Institute on Aging-Alzheimer’s Association^77^. Dementia severity at the time of blood collection was determined using the CDR. Controls without dementia underwent the same assessments and maintained a CDR score of 0. Participants in this study were classified into different disease categories or as healthy controls based on their clinical diagnosis.

### Differential abundance analysis

To identify proteins associated with each ND, we performed differential abundance analysis using linear regression models comparing clinical cases (AD, PD and FTD) to cognitively normal controls. Controls served as the reference group for all comparisons. Analyses for amyotrophic lateral sclerosis were not performed because the data came from only two contributors that lacked matched control participants. Linear regression models were constructed using the ‘lm’ function from the base ‘stats’ package in R (version 4.4.0)^78^, applied to the log_10_-transformed, *z*-score normalized protein aptamer data.

For each disease, we adjusted for common confounding variables, including age, sex and the first two proteomic principal components, except in the case of FTD, where sex information was unavailable from one contributing site at the time of analysis. To assess the impact of missing sex information, we conducted a sensitivity analysis using only samples with complete sex and age data. A comparison of effect sizes between models with and without sex adjustment revealed a strong correlation (*r*^2^ = 0.99) and a high concordance rate (96.5%), with a similar number of significantly altered proteins (13.91% with sex versus 13.44% without sex). These findings indicated that sex had a minimal effect on the results for FTD.

Proteomic principal components were computed using the ‘prcomp’ function in the base ‘stats’ R package after imputing missing protein expression values using a bootstrapping-based approach. To control for false positives, *P* values from the linear regression analysis were adjusted for FDR using the Benjamini–Hochberg method^79^, implemented via the ‘p.adjust’ function in R. Proteins with FDR < 0.05 were deemed statistically significant. The results were visualized using volcano plots generated with the EnhancedVolcano package (version 1.18.0, RRID: SCR_018931), displaying significantly upregulated and downregulated proteins for each disease.

### Odds ratio analysis

After the differential abundance analysis, we evaluated the association between protein levels and disease risk (AD, PD and FTD) using tertile-based odds ratio analysis. For each of the significantly disease-associated proteins, the highest (3rd) and lowest (1st) tertiles of protein abundance were compared using logistic regression models using the ‘stats’ package ‘glm’ function in R. Disease status (AD, PD or FTD) was modeled as the outcome, and protein tertile (1st versus 3rd), age, sex and the first two proteomic principal components were included as covariates. The results are summarized as odds ratios with 95% confidence intervals. FDR was performed using the Benjamini–Hochberg method. To evaluate overall effect size magnitudes, a symmetric odds ratio distribution was constructed by inverting odds ratios less than 1, and the mean odds ratio was computed for each disease group.

### Sensitivity tests to assess the robustness of the differential abundance analyses

For each disease group, AD, PD and FTD, only study sites that included both cases and controls were retained to ensure valid comparisons of disease status within each site. For AD, 10 sites were included: nine EDTA-based sites and one citrate-based site (Site E). For PD, five sites were included (Sites C, F, J, L and Q). For FTD, four sites were included (Sites C, I, N and Q). These same sets of sites were used consistently in both joint and by-site analyses for each disease to ensure comparability (Supplementary Tables 2 and 8).

### By-site differential abundance analysis and meta-analysis

To estimate disease-associated changes in protein levels across the selected sites, by-site analysis was conducted. For each included site, the same covariate-adjusted model was applied (*z*-score normalized by entire dataset):

$${\rm{Protein}} \sim {\rm{status}}+{\rm{age}}+{\rm{sex}}+{\rm{PC}}1+{\rm{PC}}2$$

These models were fit independently within each site, and the resulting effect sizes and standard errors were then combined across sites using random effects meta-analysis with restricted maximum likelihood (REML) estimation (via the ‘rma()’ function in the ‘metafor’ R package). FDR correction was applied to the meta-analytic *P* values across all proteins.

### Joint analysis (fixed-effect size)

The joint analysis was performed by combining all eligible samples and fitting the following linear model for each protein (*z*-score normalized by entire dataset). To address potential confounding arising from inter-site variability, site was explicitly included as a covariate in the joint model:

$${\rm{Protein}} \sim {\rm{status}}+{\rm{age}}+{\rm{sex}}+{\rm{site}}+{\rm{PC}}1+{\rm{PC}}2$$

Site was included as a fixed-effect covariate, and FDR was calculated using the Benjamini–Hochberg procedure across all proteins (Supplementary Table 2i).

### Joint analysis (random-effect size)

The joint analysis was performed by combining all eligible samples and fitting the following linear model for each protein (*z*-score normalized by entire dataset). To address potential confounding arising from inter-site variability, site was explicitly included as a covariate in the joint model:

$${\rm{Protein}} \sim {\rm{status}}+{\rm{age}}+{\rm{sex}}+(1\,|\,{\rm{site}})+{\rm{PC}}1+{\rm{PC}}2$$

Site was included as a random intercept, and FDR was calculated using the Benjamini–Hochberg procedure across all proteins (Supplementary Table 2i).

### EDTA versus sodium citrate

To evaluate the impact of including the citrate-based cohort in joint differential abundance analysis, two models were compared: one using only EDTA-based samples and another combining EDTA and citrate samples. For both models, *z*-score transformation of protein abundance was performed across all samples after quality control, using the same linear regression model:

$${\rm{Protein}} \sim {\rm{status}}+{\rm{age}}+{\rm{sex}}+{\rm{PC}}1+{\rm{PC}}2$$

Differential abundance analysis was performed without stratification by site for AD versus controls. Differentially expressed proteins were defined based on FDR < 0.05 using the Benjamini–Hochberg procedure. Concordance between the two joint analyses was assessed based on effect size direction, nominal and FDR significance and Pearson’s correlation (Supplementary Table 2c,d).

### Orthogonal validation

To assess the consistency of protein–disease associations from the GNPC (SomaScan platform) across other datasets and platforms, we compared our results with the UK Biobank^23^ and additional samples from the Stanford and Knight ADRC cohorts. The comparison with the UK Biobank uses Olink for PD, and the comparison with the Knight ADRC uses Alamar, which are different platforms than the GNPC, which would represent an orthogonal validation. The comparison with the Stanford samples could represent a replication. The PD comparison used data from Rutledge et al.^11^ that included SomaScan measurements from 652 controls and 429 PD samples. Finally, we used Alamar data from 1,579 controls and from 1,092 AD and 39 FTD cases (Supplementary Table 2g,h).

### Pathway analysis

To investigate the biological processes associated specifically with each ND and those common to all, we performed pathway enrichment analysis using the ReactomePA (version 1.48)^80^ and clusterProfiler (version 4.8.1) R packages^81^. The analysis was conducted separately for disease-specific proteins unique to each ND and for proteins overlapping across multiple NDs. Disease-specific proteins were defined as those significantly associated with only one disease (FDR < 0.05) and not with others.

Pathway enrichment was performed using Reactome^82^ pathways through the ‘enrichPathway’ function, with the default reference background. A hypergeometric test was used to assess pathway overrepresentation, followed by multiple comparison correction using the FDR method, unless specified otherwise. For pathways associated with disease-specific (uniquely associated to AD, PD or FTD) proteins, as well as those commonly altered in both PD and FTD, results were reported without applying FDR correction due to the limited number of enriched pathways.

### Cell type enrichment analysis

We applied cell type enrichment analysis to determine the specificity of aptamers from the SomaScan assay version 4.1 for various human brain cell types. This analysis incorporated gene expression data from five distinct human brain cell types^83^, including astrocytes, neurons, oligodendrocytes, microglia/macrophages, endothelial cells and mature astrocytes. We averaged the gene expression levels within each cell type and calculated the sum across all cell types for each gene. From these totals, we determined each cell type’s contribution proportion. Genes were labeled as cell type specific when the expression in the highest-expressing cell type was at least 1.5 times greater than that in any other cell type. We performed a hypergeometric test using the ‘phyper’ function from the ‘stats’ package in R to evaluate the enrichment of different brain cell types in proteins associated with each disease and those participating in each biological pathway identified in the pathway enrichment analysis. The results were visualized using a heatmap of significance values (hypergeometric *P*), created with the ggplot2 R package (version 3.5.1, RRID: SCR_014601)^84^.

The above-mentioned transcriptomic study did not profile the detailed blood cell types from the human brain. To check the blood cells in the cell type enrichment analysis, we used a new dataset combined from two recent single-nuclei RNA sequencing datasets^85,86^ containing over 700 participants. We computed the cell type proportion of each gene using the pseudobulk strategy across all blood-related cell types: endothelial cells, pericytes, smooth muscle cells, natural killer cells, CD8 T cells, neutrophils, erythrocytes, fibroblasts and vascular leptomeningeal cells. To define whether the protein was cell type specific, we required its corresponding gene expression to be at least half-fold higher in one cell type compared to any other cell type. To test for the enrichment, we calculated the odds ratio via Fisher’s exact test for each cell type within each locus. The significance threshold of the cell type being enriched was *P* < 0.05.

### Protein network analysis

Most existing network-based approaches primarily rely on protein co-expression networks, which capture correlations in protein abundance rather than direct molecular interactions. Incorporating actual PPI data could provide a more accurate representation of disease-related biological processes and uncover mechanistic insights that may be missed by co-expression analyses alone. To analyze shared protein dysregulation and their interactions across AD, PD and FTD, we created a PPI network for proteins that are significantly associated (FDR < 0.05) with all three diseases. We extracted interactions that are curated from multiple sources in STRING (version 12.0, accessed on 4 April 2025)^87^: ‘experimental evidence’, ‘databases’, ‘co-expression’, ‘neighborhood’, ‘gene fusion’ and ‘co-occurrence’. The minimum evidence score from these multiple sources was set to 0.7 for high confidence in the observed interactions. Disconnected proteins and isolated pairs were excluded to focus on highly connected proteins. First, we generated a PPI network of all disease-associated proteins to examine global connectivity patterns (Extended Data Fig. 6a). We then narrowed our focus to proteins commonly altered across diseases and enriched in converging biological pathways (Extended Data Fig. 6b), thereby identifying biologically meaningful subnetworks that are more likely to contribute to shared common inflammation-related and vascular-disruption-related mechanisms (Extended Data Fig. 6c–i).

The disease-specific protein regulatory networks were built using a dedicated network reconstruction algorithm that relies on Boolean formalism^88^. This approach eliminates non-specific interactions from the initial set of PPIs obtained from STRING, which are inconsistent with the binarized protein expression states (proteins with increased or decreased levels in disease represented as 1 or 0, respectively) within a genetic algorithm-based optimization procedure. Because the STRING database lacks information regarding directionality of interactions (activating or inhibiting effect), the algorithm automatically infers this missing information from the Booleanized protein expression data and the known network topology.

To prioritize key upstream regulators based on their potential impact on downstream network targets, we performed an in silico network perturbation analysis^88^. This approach simulates the effect of individually perturbing candidate regulator proteins (perturbagens) and evaluates their capacity to revert Booleanized protein expression state. Perturbagens were ranked by perturbation scores, which reflect the number of downstream target proteins whose expression states are predicted to flip upon simulated regulator modulation. Higher scores indicate a greater potential to reverse expression state of more downstream protein targets, thereby identifying regulators with strong influence over the disease-specific network architecture.

We also reconstructed a non-directional STRING-based PPI network for all the disease-associated proteins. To further understand how the protein dysregulation is involved in known biological pathways, which can help uncover potential therapeutic targets in downstream analyses, we created a second network focusing on proteins that are involved in pathways of interest. We generated subnetworks to explore how protein families of interest interact with the dysregulated proteins contributing to pathway enrichment. For all network analyses, when a protein was measured by multiple aptamers, we selected the aptamer with the lowest FDR value across all three diseases to represent that protein. For all networks, we used the same interaction sources from STRING as above, and the networks were visualized using the Cytoscape (version 3.10.3) tool^89^, with proteins represented as nodes and interactions as edges.

### LASSO regression for disease-specific predictive modeling

To develop disease-specific prediction models, we identified protein aptamers that exhibited significant alterations in cases (AD, PD and FTD) compared to controls using differential abundance analysis. To minimize redundant features, proteins with highly correlated expression levels (*r*^2^ > 0.8) across all samples within each disease group were identified, and one representative protein was retained while the other was excluded from every highly co-related pair. We applied LASSO regression using the ‘glmnet’ function from the ‘glmnet’ R package (version 4.1.7) to identify the most informative set of proteins. The LASSO model was run for 50 iterations, with data randomly split into 70% training and 30% testing sets. The ‘cv.glmnet’ function was used to perform 10-fold cross-validation, determining the optimal lambda regularization parameter for each model. Across these iterations, LASSO models identified a minimum of 134 proteins in FTD to a maximum of 531 proteins in AD (Supplementary Table 9). Based on the selection frequency of proteins across all iterations, we identified the top 10 proteins that appeared most consistently across different LASSO models. These 10-protein sets were designated as disease-specific proteomic signatures and were used to train the prediction models for each disease separately using the ‘glm’ function in the base ‘stats’ R package (Supplementary Table 10).

To evaluate the predictive power of the identified disease-specific proteomic signatures, we used a two-stage approach consisting of 100 iterations of model training and testing. In each iteration, models were trained using 70% of the samples and applied to the remaining 30% using the training-derived weights. This iterative approach was chosen to prevent model overfitting caused by class imbalance in certain diseases, where the number of cases is relatively low compared to controls (for example, FTD and PD). During each iteration, 70% of the cases and an equal number of randomly selected controls were used for training, and the remaining 30% of cases and an equal number of controls were used for testing. The final model performance was reported as the average across iterations with 95% confidence intervals.

Model performance was assessed using a baseline model incorporating only age and sex as well as a model that included both the baseline variables and the identified proteomic signature. Additionally, we tested each disease-specific model against other NDs to assess cross-disease specificity. ROC curves and AUC values were generated using the ‘pROC’ R package (version 1.18.2)^90^. Sensitivity, specificity, false-positive rate, positive predictive value and negative predictive value were calculated using the ‘coords’ function in ‘pROC’, with optimal cutoffs determined using Youden’s *J* statistic^91^.

To assess the consistency of disease-specific prediction models above, we implemented a leave-one-site-out cross-validation (LOOCV) strategy using LASSO logistic regression, in which we are using the same ‘glmnet’ function (version 4.1.7) in R as the LASSO prediction model for AD, PD and FTD. For each iteration, data from one site were held out as an independent test set, and the remaining sites’ data were used to train the model. This process was repeated such that each contributor served as the test set exactly once. For each disease group, only sites with sufficient numbers of both case and control samples were included, resulting in different numbers of contributors and total sample sizes for AD, PD and FTD analyses.

To ensure consistent scaling across features while preventing data leakage, we applied site-specific *z*-score normalization to all protein measurements. That is, protein expression values were standardized within each site independently, using the mean and s.d. computed only from samples belonging to the same site. After normalization, missing values in protein features were imputed within each training fold using a bootstrap sampling strategy. Specifically, missing entries were replaced with values randomly sampled from the non-missing values of the same protein within the training data. Test set imputation was performed separately, using the same procedure but restricted to the value distribution from the training set only. This ensures that no information from the held-out site was used during model training or preprocessing.

Input features for all models included all quantified proteins, age at visit and sex. Model performance was evaluated using the area under the ROC curve, with 95% confidence intervals estimated using DeLong’s method^92^. To summarize predictive performance across contributors, we report the AUC from a previously published reference model, the per-site AUCs and a sample-size-weighted mean AUC, along with the number of test samples per site.

### Reporting summary

Further information on research design is available in the Nature Portfolio Reporting Summary linked to this article.

## Online content

Any methods, additional references, Nature Portfolio reporting summaries, source data, extended data, supplementary information, acknowledgements, peer review information; details of author contributions and competing interests; and statements of data and code availability are available at 10.1038/s41591-025-03833-1.

## Supplementary information

Supplementary Information List of GNPC members.

Reporting Summary

Supplementary Tables 1–10.

## Data Availability

The harmonized GNPC data used to generate these findings were provided to consortium members in June 2024 and will be made available for public request by the AD Data Initiative by 15 July 2025. Members of the global research community will be able to access the metadata and place a data use request via the AD Discovery Portal (https://discover.alzheimersdata.org/). Access is contingent upon adherence to the GNPC Data Use Agreement and the Publication Policies. The GNPC V1 harmonized data set (HDS) request link can be found on the GNPC website (https://www.neuroproteome.org/harmonized-data-set-hds).

## Code availability

All scripts used for data analysis are available at GitHub, publicly accessible using following weblink: https://github.com/NeuroGenomicsAndInformatics/NatMed_2025_GNPC.
