# Author Correction: Shared and disease-specific pathways in frontotemporal dementia and Alzheimer’s and Parkinson’s diseases

**DOI:** 10.1038/s41591-025-03970-7

**Published:** 2025-09-02

**Authors:** Muhammad Ali, Buddhiprabha Erabadda, Yike Chen, Ying Xu, Katherine Gong, Menghan Liu, Alexa Pichet Binette, Jigyasha Timsina, Daniel Western, Chengran Yang, Gyujin Heo, Jacob W. Vogel, Betty M. Tijms, Varsha Krish, Farhad Imam, The Global Neurodegeneration Proteomics Consortium (GNPC), Muhammad Ali, Yike Chen, Ying Xu, Menghan Liu, Alexa Pichet Binette, Jigyasha Timsina, Gyujin Heo, Jacob W. Vogel, Betty M. Tijms, Varsha Krish, Farhad Imam, Oskar Hansson, Laura Winchester, Carlos Cruchaga, Oskar Hansson, Laura Winchester, Carlos Cruchaga

**Affiliations:** 1 Department of Psychiatry, Washington University School of Medicine, St. Louis, MO USA; 2 NeuroGenomics and Informatics Center, Washington University School of Medicine, St. Louis, MO USA; 3 Department of Psychiatry, University of Oxford, Oxford, UK; 4 Clinical Memory Research Unit, Department of Clinical Sciences Malmö, Lund University, Lund, Sweden; 5 Department of Clinical Sciences Malmö, SciLifeLab, Lund University, Lund, Sweden; 6 Alzheimer Center Amsterdam, Neurology, Vrije Universiteit Amsterdam, Amsterdam UMC location VUmc, Amsterdam, The Netherlands; 7 Amsterdam Neuroscience, Neurodegeneration, Amsterdam, The Netherlands; 8 Gates Ventures, Seattle, WA USA; 9 Eli Lilly, Stockholm, Sweden; 10 Department of Neurology, Washington University School of Medicine, St. Louis, MO USA; 11 Hope Center for Neurological Disorders, Washington University, St. Louis, MO USA; 12 Department of Genetics, Washington University School of Medicine, St. Louis, MO USA

**Keywords:** Molecular neuroscience, Alzheimer's disease, Diagnostic markers

Correction to: *Nature Medicine* 10.1038/s41591-025-03833-1, published online 15 July 2025.

This article was originally published under standard Springer Nature license (© The Author(s), under exclusive licence to Springer Nature America, Inc.). It is now available as an open-access paper under a Creative Commons Attribution 4.0 International license, © The Author(s). The error has been corrected in the HTML and PDF versions of the article.

