# Supplementary material for: Shared and disease-specific pathways in frontotemporal dementia and Alzheimer’s and Parkinson’s diseases
Source: Nat Med. 2025 Jul 15;31(8):2567–77. doi: 10.1038/s41591-025-03833-1 (PMC12404994; doi:10.1038/s41591-025-03833-1)
Supplement: Supplementary file 2 — Reporting Summary [file 41591_2025_3833_MOESM2_ESM.pdf]

nature portfolio

Corresponding author(s): Carlos Cruchaga

Last updated by author(s): Apr 18, 2025

Reporting Summary

Nature Portfolio wishes to improve the reproducibility of the work that we publish. This form provides structure for consistency and transparency in reporting. For further information on Nature Portfolio policies, see our Editorial Policies and the Editorial Policy Checklist.

Statistics

For all statistical analyses, confirm that the following items are present in the figure legend, table legend, main text, or Methods section.

n/a	Confirmed	
☐	☒	The exact sample size (*n*) for each experimental group/condition, given as a discrete number and unit of measurement
☐	☒	A statement on whether measurements were taken from distinct samples or whether the same sample was measured repeatedly
☐	☒	The statistical test(s) used AND whether they are one- or two-sided<br>*Only common tests should be described solely by name; describe more complex techniques in the Methods section.*
☐	☒	A description of all covariates tested
☐	☒	A description of any assumptions or corrections, such as tests of normality and adjustment for multiple comparisons
☐	☒	A full description of the statistical parameters including central tendency (e.g. means) or other basic estimates (e.g. regression coefficient) AND variation (e.g. standard deviation) or associated estimates of uncertainty (e.g. confidence intervals)
☐	☒	For null hypothesis testing, the test statistic (e.g. *F*, *t*, *r*) with confidence intervals, effect sizes, degrees of freedom and *P* value noted<br>*Give P values as exact values whenever suitable.*
☒	☐	For Bayesian analysis, information on the choice of priors and Markov chain Monte Carlo settings
☒	☐	For hierarchical and complex designs, identification of the appropriate level for tests and full reporting of outcomes
☐	☒	Estimates of effect sizes (e.g. Cohen's *d*, Pearson's *r*), indicating how they were calculated

Our web collection on statistics for biologists contains articles on many of the points above.

Software and code

Policy information about availability of computer code

Data collection	No software was used for data collection.
Data analysis	All data analysis was performed using R (v4.4.0). R packages used include base R package (v4.4.0), EnhancedVolcano (v1.18.0), pROC (v1.18.2), ClusterProfiler (v4.8.1), ReactomePA (v1.48), glmnet (v4.1.7), and ggplot2 (v3.5.1).<br>Additionally, protein-protein interaction (PPI) information was obtained from STRING database version v12.0 accessed on 04/04/2025 and Cytoscape tool (version 3.10.3) was used to visualized the protein-protein interaction networks.<br><br>Code Availability: All scripts used for data analysis are available at Github, publicly accessible using following weblink: https://github.com/NeuroGenomicsAndInformatics/NatMed_2025_GNPC.

For manuscripts utilizing custom algorithms or software that are central to the research but not yet described in published literature, software must be made available to editors and reviewers. We strongly encourage code deposition in a community repository (e.g. GitHub). See the Nature Portfolio guidelines for submitting code & software for further information.

Data

Policy information about availability of data

All manuscripts must include a data availability statement. This statement should provide the following information, where applicable:

- Accession codes, unique identifiers, or web links for publicly available datasets
- A description of any restrictions on data availability
- For clinical datasets or third party data, please ensure that the statement adheres to our policy

The harmonized GNPC data used to generate these findings was provided to Consortium Members in June 2024 and will be made available for public request by the AD Data Initiative by July 1, 2025. Members of the global research community will be able to access the metadata and place a data use request via the AD Discovery Portal (https://discover.alzheimersdata.org/). Access is contingent on adherence to the GNPC Data Use Agreement and the Publication Policies.

Research involving human participants, their data, or biological material

Policy information about studies with human participants or human data. See also policy information about sex, gender (identity/presentation), and sexual orientation and race, ethnicity and racism.

Reporting on sex and gender	Sex was used as a covariate in the linear regression model for performing the protein differential abundance analysis. Table 1 provides information about the sex of participants from each disease and control samples.
Reporting on race, ethnicity, or other socially relevant groupings	Not relevant for the current study.
Population characteristics	Table 1 in the main manuscript summarizes basic demographic information of study participants. For each clinically defined disease group and cognitively normal control participants, we report sample size (N), percentage of females, mean age and its standard deviation (SD), and percentage of APOE4+ participants.
Recruitment	The Global Neurodegeneration Proteomics Consortium (GNPC) version 1 (V1) consists of 23 independent sites contributing plasma proteomic samples from individuals with a range of clinical backgrounds, including Alzheimer's disease (AD), Parkinson's disease (PD), Frontotemporal dementia (FTD), Amyotrophic lateral sclerosis (ALS), diabetes, cancer, and cognitively normal controls. The broader GNPC resource includes a total of 31,111 samples from 21,979 individuals diagnosed with a range of diseases, including neurodegenerative disorders, depression, diabetes, stroke, and several others. Among these, 1,638 individuals were assigned a dementia diagnosis based on a Clinical Dementia Rating (CDR®) greater than 0.5 or a Mini-Mental State Examination (MMSE) score below 19, in the absence of a confirmed clinical diagnosis. For this study, we focused on participants diagnosed with three major neurodegenerative diseases (AD, PD, and FTD) as well as healthy controls. A total of 10,527 cross-sectional plasma samples (AD = 1,936; Dementia = 1,638; PD = 525; FTD = 163; Controls = 6,265) from 16 independent contributor sites were analyzed. Plasma samples were generally collected via blood draw in the morning or mid-day, typically without requiring participants to fast. All samples underwent standardized preparation and processing protocols and were stored at -80°C until proteomic profiling.
Ethics oversight	The ethics committee of all sites who have contributed proteomic data to GNPC approved this study. Ethics approval for each individual site was obtained from their respective Institutional Review Boards (IRB), and the research was conducted following the approved protocols (WUSTL IRB approval 201109148). Written informed consent was obtained from participants or their family members, and the study design was approved by all participating institutions.

Note that full information on the approval of the study protocol must also be provided in the manuscript.

Field-specific reporting

Please select the one below that is the best fit for your research. If you are not sure, read the appropriate sections before making your selection.

☒ Life sciences ☐ Behavioural & social sciences ☐ Ecological, evolutionary & environmental sciences

For a reference copy of the document with all sections, see nature.com/documents/nr-reporting-summary-flat.pdf

Life sciences study design

All studies must disclose on these points even when the disclosure is negative.

Sample size	Table 1 summarized the sample sizes of each cohort. No power analysis for sample size was performed . The selection of sample size (N = 10,527) was based on past studies, where N ~ 1,000 was sufficient to discover significantly differentially abundant protein aptamers.
Data exclusions	First, we applied log10 transformation to the RFU proteomic values to approximate a normal distribution. Outliers were then identified using inter-quartile range (IQR) based statistical approach. Any proteomic measure that was lower than Q1-1.5*IQR and higher than Q3+1.5*IQR, where Q1 and Q3 are the first and the third quartiles, respectively, were marked as outliers and set to missing (Fig. S7). A 65% call rate threshold was then applied such that any sample or analyte that had a call rate lower than 65% were removed from the matrix. This was followed by recalculation of call rate and second pass removal of analytes and samples using a stringent 85% call rate. Finally, analytes

targeting non-human proteins or those missing proper annotations were removed.

Replication	The broader GNPC resource includes a total of 31,111 samples from 21,979 individuals diagnosed with a range of diseases, including neurodegenerative disorders, depression, diabetes, stroke, and several others. Among these, 1,638 individuals were assigned a dementia diagnosis based on a Clinical Dementia Rating (CDR®)24 greater than 0.5 or a Mini-Mental State Examination (MMSE)25 score below 19, in the absence of a confirmed clinical diagnosis. For this study, we included only well-characterized and deeply phenotyped individuals with clinical diagnoses of AD, FTD, PD and cognitively normal controls. Individuals categorized as dementia based on clinical tests but without confirmed AD diagnosis were not included in the main analyses, although additional sensitivity analyses were performed including AD and dementia participants.
Randomization	All association tests were controlled by age, sex, and first two proteomic principal components (PC1 and PC2) to account for data variation.
Blinding	Not relevant for the current study.

Reporting for specific materials, systems and methods

We require information from authors about some types of materials, experimental systems and methods used in many studies. Here, indicate whether each material, system or method listed is relevant to your study. If you are not sure if a list item applies to your research, read the appropriate section before selecting a response.

Materials & experimental systems

n/a	Involved in the study
☒	☐ Antibodies
☒	☐ Eukaryotic cell lines
☒	☐ Palaeontology and archaeology
☒	☐ Animals and other organisms
☒	☐ Clinical data
☒	☐ Dual use research of concern
☒	☐ Plants

Methods

n/a	Involved in the study
☒	☐ ChIP-seq
☒	☐ Flow cytometry
☒	☐ MRI-based neuroimaging

Plants

Seed stocks	Not relevant for the current study.
Novel plant genotypes	Not relevant for the current study.
Authentication	Not relevant for the current study.
